# Perceived school-wide teacher-student relations and teacher job satisfaction: An affective events theory analysis of Shanghai teachers

**DOI:** 10.1371/journal.pone.0357279

**Published:** 2026-09-03

**Authors:** Mengting Qian, Chunshun Yan, Xinyi Wang

**Affiliations:** 1 Faculty of Education, East China Normal University, Shanghai, China; 2 Berkeley School of Education, University of California, Berkeley, California, United States of America; 3 Ontario Institute for Studies in Education, University of Toronto, Toronto, Ontario, Canada; Golestan University, IRAN, ISLAMIC REPUBLIC OF

## Abstract

Teachers’ perceptions of teacher-student relations in their school are linked to their job satisfaction, but whether positive teaching-related emotion constitutes a statistical indirect association between them, and whether school leadership context alters it, remains unclear. Using Teaching and Learning International Survey (TALIS) 2024 data for Shanghai (China), this study analyzed the OECD scales for teacher-student relations, joy of teaching, job satisfaction, and principal relational leadership in 1,338 teachers from 205 schools. A conditional process regression adjusted for teacher demographic and career characteristics; point estimates used the final teacher weight, and sampling variance was estimated from 100 Fay balanced repeated replication weights. Perceived school-wide teacher-student relations were positively associated with job satisfaction, and the indirect association through joy of teaching was supported. Principal relational leadership did not significantly alter the conditional direct association, whereas its interaction with joy of teaching was positive; this interaction was not reproduced when the constructs were scored as simple item means, so the moderation result is reported as exploratory. The pattern is consistent with the work environment-emotion-work attitude linkage proposed by Affective Events Theory. Conclusions are restricted to lower-secondary teachers in Shanghai and offer leads for longitudinal and intervention research.

## 1. Introduction

Teacher job satisfaction reflects teachers’ overall evaluation of their professional role, working conditions, and work experiences, and it is a central indicator of teacher well-being and school functioning [1,2]. Low job satisfaction has been linked to burnout, impaired health, reduced career persistence, and weaker instructional quality [3,4]. Identifying the classroom experiences, teaching emotions, and organizational conditions reliably associated with job satisfaction therefore has clear theoretical and practical value.

Correlates of teacher job satisfaction span individual characteristics, working conditions, and the organizational environment [5,6]. Within daily instructional work, the interactions teachers have with students and the relational climate those interactions create are particularly consequential [7]. Large-scale international surveys have repeatedly shown that more positive perceptions of teacher-student relations accompany higher job satisfaction [8,9], and positive relational experience has also been associated with professional identity, instructional efficacy, and work motivation [10,11]. Because the relevant survey items ask teachers to evaluate the general state of teacher-student relations in their school rather than a specific dyad, this study defines the construct as teachers’ perceived school-wide teacher-student relational climate.

Joy of teaching is a plausible emotional link between this climate and teachers’ work attitudes. It refers to the positive emotion teachers experience during instructional activity [12,13]. More positive interactions with students accompany greater joy of teaching, which is in turn related to work engagement and occupational well-being [14,15], and teacher and student emotions may be reciprocally coupled over time [16,17]. This evidence justifies testing whether joy of teaching constitutes an indirect association between the relational climate and job satisfaction.

School leadership is a salient organizational context for teachers’ work experience. Existing studies report that distributed leadership is related to teacher commitment and job satisfaction, that teacher leadership is related to teacher well-being, and that principals’ social support is related to emotional exhaustion [18–21], all constructs adjacent to but not identical with relational leadership. From the perspective of Affective Events Theory (AET), the organizational context may be directly related to teachers’ evaluation of their work and may also alter the strength of the association between positive emotion and work attitudes [22]. Where relational support from the principal is strong, joy of teaching may be more likely to accompany favorable appraisals of the work environment; where such support is weak, an equivalent level of joy of teaching need not correspond to equally high job satisfaction.

Three questions remain insufficiently resolved. First, teacher-student relations, teacher emotion, and job satisfaction are frequently examined separately, and joint tests of the three constructs remain limited. Second, whether principal leadership relates to the conditional direct association between teacher-student relations and job satisfaction, or primarily to the conditional association between joy of teaching and job satisfaction, needs to be distinguished within a single model. Third, TALIS data involve questionnaire rotation, the nesting of teachers within schools, and a complex sampling design; ignoring planned non-administration, sampling weights, and design-based variance can bias standard errors and inference.

The present study therefore uses TALIS 2024 data for teachers at level 2 of the International Standard Classification of Education (ISCED 2) in Shanghai (China) to test the adjusted association between the perceived school-wide relational climate and job satisfaction, to test whether joy of teaching constitutes a statistical indirect association between them, and to use a regression specification corresponding to Model 15 of Hayes [23] to test separately whether principal relational leadership alters the conditional direct association between the relational climate and job satisfaction and the second-stage association between joy of teaching and job satisfaction. AET provides the theoretical frame, and both the study population and the scope of inference are restricted to lower-secondary teachers in Shanghai.

## 2. Theoretical framework and hypotheses

### 2.1. Affective events theory

AET explains the linkages among work events, individual emotion, and work attitudes [22]. It distinguishes the work environment, discrete work events, emotional reactions, and comparatively stable work attitudes: appraisals of events are related to emotional experience, recurring emotional experience is related to global attitudes such as job satisfaction, and individual characteristics and organizational context may alter the strength of these linkages. Meta-analytic work has further shown that associations between discrete emotions and job satisfaction are not uniform across emotion types [24].

In educational settings, teacher-student interaction is a frequent and emotionally meaningful source of experience [25,26]. Teachers continuously appraise student participation, classroom discipline, goal attainment, and relational quality, and these appraisals are linked to enjoyment, anxiety, and anger during teaching [27]. More positive perceptions of teacher-student interaction likewise co-occur with greater joy of teaching, and teacher emotions relate to teaching quality and job satisfaction [14,28]. Leaders’ emotional competence and supportive behavior have also been associated with teacher well-being [29].

**Fig 1:**
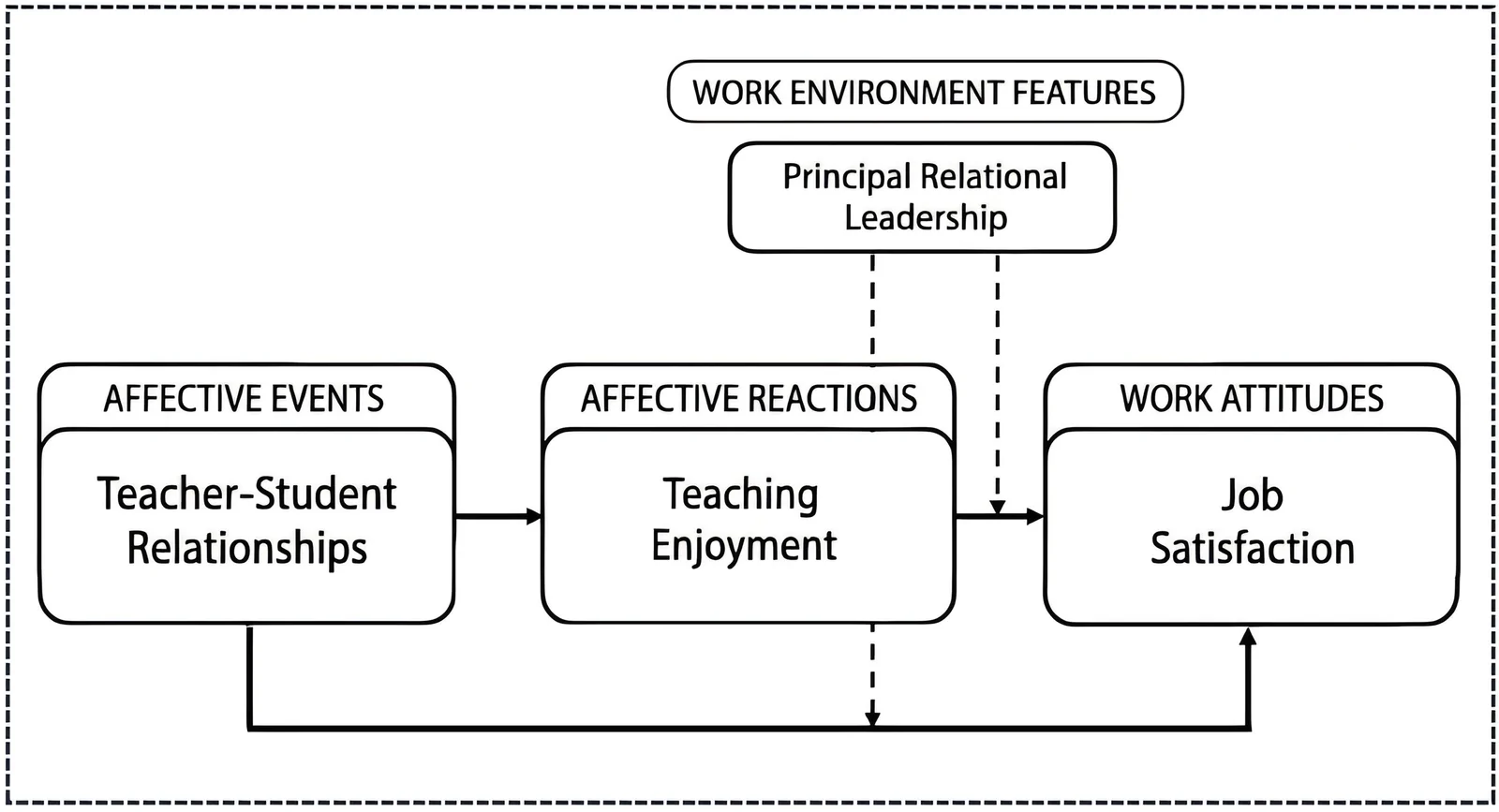
Conceptual mapping of the study variables onto Affective Events Theory. Alt text: A conceptual Affective Events Theory diagram links perceived school-wide teacher–student relations, joy of teaching, and teacher job satisfaction, with principal relational leadership shown as a contextual condition.

In the present study, the TALIS indicator of teacher-student relations corresponds to the broader work-relationship environment in AET; joy of teaching corresponds to positive instructional emotion; job satisfaction corresponds to a global work attitude; and perceived principal relational leadership corresponds to an organizational contextual condition. These theoretical linkages are summarized in Fig 1, in which the arrows denote theory-specified regression associations rather than identified causal or temporal effects.

### 2.2. Relations, emotion, and leadership in the Shanghai school context

Teacher-student relations in Chinese schools are shaped both by institutional arrangements and by a cultural tradition that accords high value to the teacher’s role. Research suggests that the tradition of respect for teachers may support teacher authority and relational continuity while also constraining certain forms of egalitarian communication [30]. Teachers in this system also face multiple job demands, including instructional workload, examination pressure, home-school communication, and policy change [31], and studies of teachers in different regions of China have reported substantial occupational stress and burnout risk [32]. The linkage between positive emotions such as joy of teaching and global job satisfaction therefore merits closer examination in high-demand work environments.

Principal leadership is one contextual factor for understanding variation in teacher job satisfaction [33]. Distributed leadership is related to school and teacher characteristics [34] and to teachers’ social and emotional learning practices and job satisfaction [20,35], indicating that school organization and leadership context are both relevant to teachers’ work experience [36]. This study measures teachers’ perceptions of principal relational leadership, so the conditional associations examined here operate at the teacher level.

### 2.3. Hypotheses

Teacher-student relations typically encompass respect, support, communication, and interaction quality [37]. A more positive school-wide relational climate implies that student well-being is valued and that support is available when students need extra help, and it may be linked to teachers’ sense of professional meaning, belonging, and instructional efficacy [11,38]. Persistent relational strain, by contrast, tends to co-occur with emotional exhaustion and lower occupational well-being [39]. Reviews, longitudinal studies, and school climate research converge in showing stable associations among teachers’ relational connection with students, perceived school climate, and occupational well-being [8,9,38,40].

H1: After adjustment for the predetermined background variables, teachers’ perceived school-wide teacher-student relational climate is positively associated with teacher job satisfaction.

A more positive relational climate generally implies more supportive classroom interaction and more favorable student feedback, and high-quality teacher-student interaction has been linked to teachers’ positive emotions and sense of accomplishment [10,15,41].

H2: After adjustment for the predetermined background variables, teachers’ perceived school-wide teacher-student relational climate is positively associated with joy of teaching.

AET distinguishes emotional experience from global work attitudes while proposing that the two are persistently related [22]. Teachers who frequently experience joy of teaching tend to appraise instructional activity more favorably and to perceive greater meaning in their work [42], and studies of positive affect and job satisfaction, together with meta-analytic evidence on discrete emotions, support a positive association between the two [14,24].

H3: After adjustment for teachers’ perceived school-wide teacher-student relational climate and the predetermined background variables, joy of teaching is positively associated with teacher job satisfaction.

H2 and H3 jointly constitute an indirect pathway consistent with AET: a more positive relational climate is related to greater joy of teaching, which is in turn related to higher job satisfaction.

H4: Teachers’ perceived school-wide teacher-student relational climate has a positive statistical indirect association with teacher job satisfaction through joy of teaching.

Relational leadership emphasizes the trust, respect, participation, and supportive relationships that develop between leaders and organizational members through sustained interaction [43], and leadership outcomes are themselves constrained by the organizational context [44]. Where principals value teacher participation, maintain open communication, and cultivate sound professional relationships, teachers may develop a stronger sense of organizational support [19,21].

Two conditional arguments follow. First, even with joy of teaching in the model, the relational climate may remain related to job satisfaction through channels not measured here, such as professional belonging or school identification. Where teachers perceive higher levels of principal relational leadership, the participatory environment of the school may be more congruent with a positive appraisal of that climate; where such perceptions are lower, the conditional direct association may be weaker.

H5: Teachers’ perceived principal relational leadership positively moderates the conditional direct association between the school-wide teacher-student relational climate and job satisfaction, such that the positive association is stronger at higher levels of perceived leadership.

Second, joy of teaching arises in specific instructional activity, whereas job satisfaction is a global evaluation of the profession and the work environment. A leadership context marked by support, recognition, and participation may render positive instructional emotion more congruent with overall work appraisal [18,20]. In Model 15 this corresponds to the *M* × *W* term in the outcome equation: if the *a* path and the *M* × *W* coefficient are both positive, the model-implied conditional indirect association *a*(*b*_1_ + *b*_3_*W*) increases with *W*, and the formal test is whether the confidence interval for the index of moderated mediation *a* × *b*_3_ excludes zero.

H6: Teachers’ perceived principal relational leadership positively moderates the association between joy of teaching and job satisfaction, such that the positive association is stronger at higher levels of perceived leadership, and the conditional indirect association linking the relational climate to job satisfaction through joy of teaching is correspondingly stronger.

**Fig 2:**
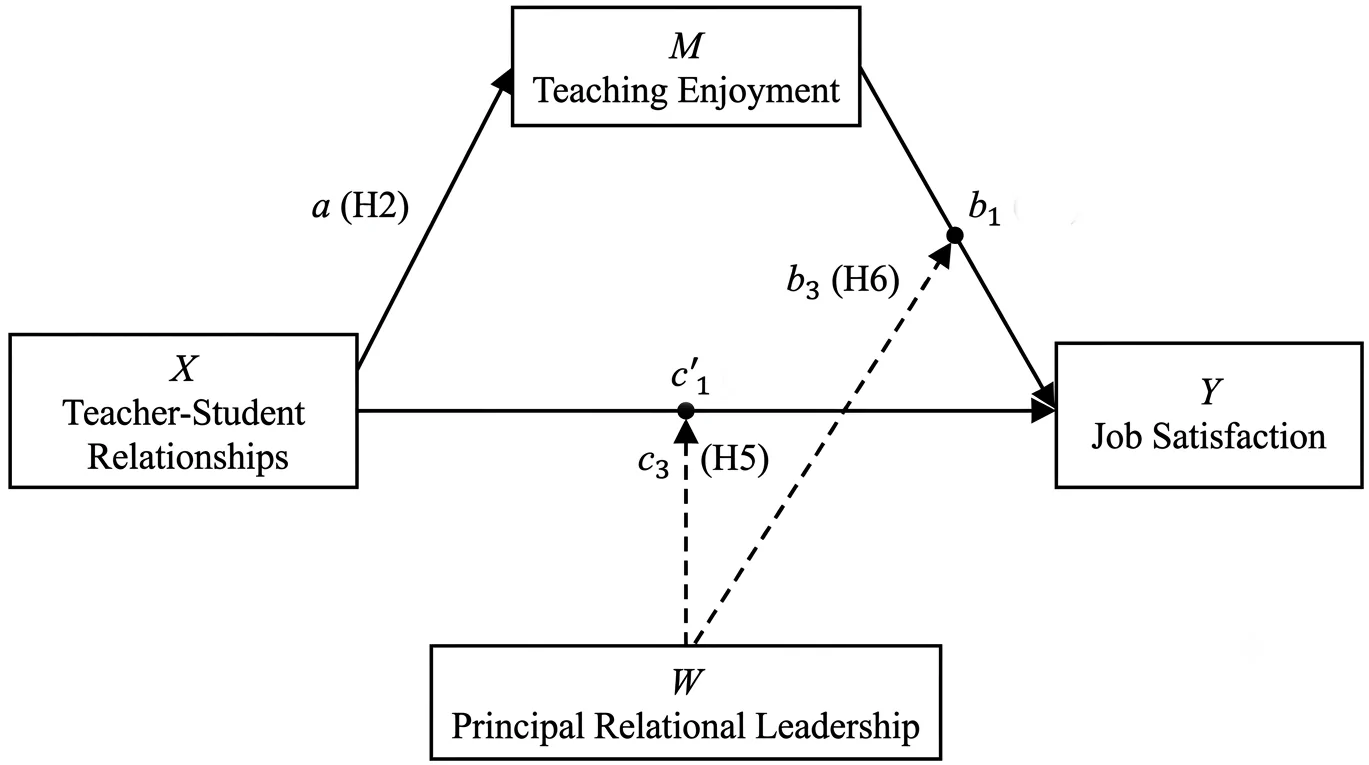
Hypothesized conditional process model corresponding to Hayes Model 15. Alt text: The research framework links perceived school-wide teacher–student relations to joy of teaching and teacher job satisfaction. Principal relational leadership conditions the direct X-to-Y association and the M-to-Y association; no first-stage moderation is specified.

Let *X* denote the perceived school-wide teacher-student relational climate, *M* joy of teaching, *Y* teacher job satisfaction, *W* perceived principal relational leadership, and *C* the vector of predetermined control variables. The study estimates


$$\begin{array}{c} M\;=\;i_{\text{M}}\;+\;aX\;+\;\gamma^{\prime }C\;+\;e_{\text{M}} \end{array}$$

$$\begin{array}{c} Y\;=\;i_{\text{Y}}\;+\;c_{\text{1}}^{\prime }X\;+\;b_{\text{1}}M\;+\;w_{\text{1}}W\;+\;c_{\text{3}}XW\;+\;b_{\text{3}}MW\;+\;\delta^{\prime }C\;+\;e_{\text{Y}} \end{array}$$

where H5 corresponds to *c*_3_ > 0 and H6 to *b*_3_ > 0 and *a* × *b*_3_ > 0. The hypothesized model is displayed in Fig 2, in which solid arrows denote hypothesized regression associations and dashed arrows denote moderating (interaction) effects.

## 3. Materials and methods

### 3.1. Data source and analytic sample

The study uses the teacher public-use file of TALIS 2024, published by the Organisation for Economic Co-operation and Development (OECD). The target population comprises ISCED 2 teachers in Shanghai (China). TALIS employs a stratified probability design with schools and teachers within schools as the two sampling stages; the analysis uses the OECD final teacher weight for point estimation and the teacher replicate weights for variance estimation, so that the estimand remains consistent with the survey design [45].

The Shanghai teacher file contains 4,078 teachers from 205 schools. TALIS 2024 uses a planned rotation of three teacher questionnaire forms: Form A was completed by 1,359 teachers, Form B by 1,355, and Form C by 1,364. The four constructs required here were administered jointly only in Form A, so the 2,719 respondents assigned to Forms B and C represent planned non-administration arising from questionnaire rotation rather than cases deleted during data cleaning. After exclusion of cases with unavailable official scale scores or incomplete control variables (see Section 3.3), the analytic sample comprised 1,338 teachers covering all 205 schools, with 4–9 teachers per school and an arithmetic mean of 6.527.

Screening was based solely on questionnaire form, availability of the official scale scores, and completeness of the predetermined controls. All raw responses fell within the prescribed range of 1–4, and no outlier deletion was performed. The derivation sequence was 4,078 teachers, then 1,359 Form A respondents, then 1,354 with all four official scale scores available, then 1,338 with complete controls. This sequence and the characteristics of the analytic sample are reported in Table 1.

**Table 1:** Derivation of the analytic sample and survey-weighted sample characteristics.

Stage or characteristic	Unweighted *n*	Schools	Notes or weighted %
***Panel A. Derivation of the analytic sample***			
Shanghai (China) ISCED 2 teacher file	4,078	205	Forms A to C; 16–29 teachers per school (*M* = 19.893)
Eligible Form A respondents	1,359	205	2,719 planned-form exclusions; 4–10 per school (*M* = 6.629)
All four official OECD scores available	1,354	205	5 unavailable official scores; 4–10 per school (*M* = 6.605)
Analytic sample	1,338	205	16 incomplete controls; 4–9 per school (*M* = 6.527)
***Panel B. Characteristics of the analytic sample***			***Weighted %***
Gender: female	984		74.194
Gender: male	354		25.806
Age: under 30 years	249		19.611
Age: 30–39 years	413		30.817
Age: 40–49 years	400		29.522
Age: 50 years or older	276		20.050
Education: ISCED 6 or below	1,038		77.182
Education: ISCED 7 or 8	300		22.818
Teaching experience: 5 years or less	269		21.171
Teaching experience: 6–10 years	243		17.809
Teaching experience: 11–20 years	317		23.455
Teaching experience: more than 20 years	509		37.565

*Note.* No cases were excluded as outliers.

This study is a secondary analysis of de-identified public-use data and involved no new recruitment of or contact with participants. Consent procedures and research governance for the original survey were the responsibility of the OECD and its national implementing agencies.

### 3.2. Measures

The primary analysis uses the scale scores generated by the OECD and distributed in the TALIS 2024 data files. Except for the composite job satisfaction scale, TALIS scales are derived from ordinal multiple-group confirmatory factor analysis parameters estimated in the pooled international sample and transformed onto a common metric. A respondent receives a scale score if at least two source items were answered, and no score is imputed for respondents who answered none. For general scales, a value of 10 corresponds to the midpoint of the item response scale and the calibration standard deviation is 2; composite scales are separately standardized to a mean of 10 and a standard deviation of 2. Because the scales are calibrated separately, their means are not directly comparable across constructs [45]. All scales are teacher self-reports, with higher scores indicating higher levels of the construct.

The independent variable was T4STUD (teacher-student relations), formed from items TT4G65A to TT4G65D, which ask whether teachers and students usually get on well, whether most teachers are interested in what students have to say and believe that student well-being is important, and whether the school provides support when students need extra help. Because the items ask teachers to judge what generally happens in their school, the construct is operationalized as the perceived school-wide relational climate rather than as a dyadic relationship (weighted Cronbach’s alpha = 0.907).

The mediator was T4JOYTCH (joy of teaching), formed from items TT4G80A to TT4G80D, which cover enjoyment of the subject taught, frequent enjoyment while teaching, habitual enthusiasm, and satisfaction derived from the challenges of teaching (weighted alpha = 0.909).

The dependent variable was T4JOBSAT (job satisfaction, overall), an OECD composite of satisfaction with the profession (T4JSPROT, from TT4G78A, TT4G78B, reverse-scored TT4G78D, and TT4G78J) and satisfaction with the work environment (T4JSENVT, from reverse-scored TT4G78C, TT4G78E, and TT4G78G). Because T4JOBSAT is a composite, an alpha across the seven source items is not an official unidimensional reliability estimate; as a within-sample description, that alpha was 0.845, and the weighted alphas for T4JSPROT and T4JSENVT were 0.765 and 0.727.

The moderator was T4RELEAD (relational leadership), formed from items TT4G66E to TT4G66H, covering the extent to which the principal encourages staff participation in important decisions and maintains sound professional relationships with staff, with parents or guardians, and with students (weighted alpha = 0.961). Measure characteristics and weighted descriptive statistics are reported in Table 2.

**Table 2:** Measures, reliability, and survey-weighted descriptive statistics and correlations.

Variable	Weighted *M*	Weighted *SD*	Weighted *α*	1	2	3	4
1. Teacher-student relations (*X*)	14.376	2.014	0.907	1.000			
2. Joy of teaching (*M*)	13.412	2.082	0.909	0.452	1.000		
3. Teacher job satisfaction (*Y*)	9.567	1.958	0.845^a^	0.453	0.599	1.000	
4. Principal relational leadership (*W*)	13.238	1.897	0.961	0.648	0.418	0.547	1.000

*Note.* ^a^Descriptive alpha across the seven source items, not an official unidimensional reliability estimate.

All models adjust for teacher gender (male indicator, female as reference), age (under 30 as reference, with indicators for 30–39, 40–49, and 50 or older, the last merging the original 50–59 and 60 or older groups), highest educational attainment (ISCED level 7 or 8 versus level 6 or below), and total teaching experience (5 years or less as reference, with indicators for 6–10, 11–20, and more than 20 years). This coding avoids treating ordered category numbers as equally spaced continuous scores and avoids unstable coefficients for very small categories. The same controls enter both the mediator and the outcome equation.

### 3.3. Planned rotation and missing data

In the source files, items not administered by design are coded 8 (9998 for the official scales), whereas administered items with omitted or invalid responses are coded 9 (9999 for the official scales). Form B did not administer TT4G65 or TT4G66, and Form C did not administer TT4G80, so the corresponding blanks constitute planned missingness rather than respondent refusal. Consistent with the rotation design, these structural non-administrations were not treated as ordinary item missingness or imputed, and the joint four-construct model was restricted to Form A.

Within Form A, official scale scores were generated under the OECD rule that at least two source items must be answered. The present study does not re-estimate or impute the official scales; the analysis excludes only cases for which an official scale score was unavailable or a control variable was incomplete. Valid scores were available for 1,355 respondents on T4STUD and for 1,356 respondents on each of T4JOYTCH, T4JOBSAT, and T4RELEAD, with an intersection of 1,354 teachers. Marginal missing counts on the controls were 7 for gender, 2 for age, 2 for educational attainment, and 11 for teaching experience; because these overlap, sequential exclusion removed 16 further teachers, yielding *N* = 1,338. Given the low proportion of ordinary missingness remaining after the design-based restriction, and given that the official scales had already been generated under the OECD scoring rule, no secondary imputation was performed. The full classification of missingness and the stepwise derivation are reported in Table S1 in S1 File, and a weighted demographic comparison of included and not-included cases is reported in Table S2 in S1 File.

The value *N* = 1,311 is not the analytic sample but a stricter subset requiring complete responses on all 18 source items and all four controls. The additional 27 teachers satisfy the official OECD scoring rule and therefore enter the primary analysis, although they do not meet item-by-item completeness. Sensitivity analyses restrict the official scales to this common sample and compare them with the item-mean specification, but the analytic sample is not redefined on that basis.

### 3.4. Analytic strategy

Analyses were carried out with reproducible scripts in R 4.6.0, using haven 2.5.5 to read the source files, survey 4.5 to construct the Fay balanced repeated replication (BRR) design and to re-estimate derived quantities within replicates, and ggplot2 4.0.3 for the interaction plot. The primary models use the official OECD scales and the *N* = 1,338 sample and adopt the conditional process regression structure of Model 15 [23] without using the PROCESS standard errors, which assume simple random sampling. All point estimates are weighted least squares estimates based on the final teacher weight. The estimated models are


$$\begin{array}{c} M_{\text{i}}\;=\;\alpha_{\text{M}}\;+\;{aX}_{\text{i}}\;+{\;\gamma^{\prime }C}_{\text{i}}\;+\;\varepsilon_{\text{Mi}} \end{array}$$

$$\begin{array}{c} Y_{\text{i}}\;=\;\alpha_{\text{Y}}\;+\;{c^{\prime }X}_{\text{i}}\;+\;{bM}_{\text{i}}\;+\;dW_{i}\;+\;{c_{\text{3}}X}_{\text{i}}W_{\text{i}}\;+\;b_{\text{3}}M_{\text{i}}W_{\text{i}}\;+\;{\delta^{\prime }C}_{\text{i}}\;+\;\varepsilon_{\text{Yi}} \end{array}$$

Here *c*_3_ tests whether relational leadership moderates the conditional direct association between *X* and *Y*, and *b*_3_ whether it moderates the association between *M* and *Y*; the second-stage index of moderated mediation is *ab*_3_. At a given value *W* = *w*, the conditional direct association is *c*′(*w*) = *c*′ + *c*_3_*w*, the conditional *M* to *Y* slope is *b*(*w*) = *b* + *b*_3_*w*, and the conditional indirect association is IE(*w*) = *a*(*b* + *b*_3_*w*). No *X* × *W* term was included in the mediator equation, so no first-stage moderation is tested or claimed. The total association *c* and the coefficients *a*, *b*, *c*′, and the product *ab* were also estimated under a Model 4 structure. Because the data are cross-sectional self-reports, *ab* and IE(*w*) are interpreted as statistical indirect associations rather than as temporally ordered causal mechanisms.

Before the product terms were constructed, *X*, *M*, and *W* were centered at their weighted means; the control indicators were not centered. Centering constants obtained in the full sample were held fixed across the 100 replicate models so that full-sample and replicate estimates refer to the same parameter. Conditional effects were probed at one weighted standard deviation below the weighted mean of W, at the weighted mean, and at one weighted standard deviation above it, corresponding to original-scale values of 11.3406, 13.2380, and 15.1354, respectively; these probe values were likewise held fixed across replicates.

Significance was evaluated with two-sided *α* = 0.05, and unstandardized coefficients, Fay-BRR standard errors, 95% confidence intervals, and exact *p* values are reported. Nonlinear derived quantities, including *ab*, *ab*_3_, and all conditional associations, were recomputed within each replicate model before their design-based variance was estimated. Moderated mediation was judged by whether the confidence interval for *ab*_3_ excluded zero, not by whether the conditional indirect associations at the three probe values were individually significant. The joint contribution of the two interaction terms was evaluated with a Wald test using the full Fay-BRR covariance matrix, and the weighted *R*² is reported only as a descriptive index of fit.

### 3.5. Survey weights, replication variance, and clustering

Point estimates for the teacher population use the final teacher weight (TCHWGT). Sampling variance was estimated from the 100 teacher Fay BRR weights (TRWGT1 to TRWGT100) with the Fay coefficient set to *ρ* = 0.5. For any parameter *θ̂*, the variance estimator is


V^BRR(θ^) = {1 / [100(1 − ρ)2]} × Σ (θ^r − θ^)2, summed over r = 1 to 100, with ρ = 0.5

where *θ̂* is the full-sample estimate based on the final weight and *θ̂*_r_ is the estimate from replicate *r*. The 100 replicate models use identical analytic cases, control coding, and centered variable definitions. The main tables construct comparatively conservative 95% confidence intervals from the *t*(99) distribution; a check using the standard normal critical value of 1.96 produced identical conclusions. Fay-BRR is the formal design-based inference, whereas weighted cluster-robust (CR1) standard errors computed across the 205 schools serve only as a sensitivity check and are not combined with the Fay-BRR standard errors.

To quantify within-school clustering, unweighted one-way random-intercept intraclass correlation coefficients, ICC(1), were computed for the four official scales, and approximate design effects were obtained as 1 + (effective mean cluster size – 1) × ICC, using an effective mean cluster size of 6.526. The ICC(1) values were 0.0571 for teacher-student relations, 0.0738 for joy of teaching, 0.0890 for overall job satisfaction, and 0.0919 for principal relational leadership, with design effects of 1.316, 1.408, 1.492, and 1.508 (Table S3 in S1 File). Because within-school correlation is clearly nonzero, treating the 1,338 teachers as fully independent observations is not appropriate for formal inference.

Multilevel modeling was not adopted as a substitute for a standard error correction. Both *X* and *W* are teacher-level subjective perceptions, and the research question concerns conditional associations at the teacher level; decomposing each teacher’s deviation from the school mean and the school mean itself would change both the parameters estimated and the meaning of the hypotheses. The analytic sample also contains only 4–9 teachers per school, which limits the aggregation reliability of school means, and an appropriate multilevel analysis of TALIS data would require explicit decisions about level-specific weight scaling and within-between decomposition. The study therefore uses the final teacher weight with Fay-BRR for formal complex-survey inference and school-level CR1 results as a sensitivity check, reporting both alongside uncorrected ordinary least squares results in Table S4 in S1 File. The results are not interpreted as school-level leadership effects.

To separate changes in sample composition from changes in scale operationalization, three specifications were compared as planned: (a) official scales with the maximum available sample, *N* = 1,338; (b) official scales restricted to the item-complete sample, *N* = 1,311; and (c) the raw item means used previously, *N* = 1,311. Satisfaction with the work environment, satisfaction with the profession, and the six-item outcome mean used previously were also substituted for overall job satisfaction, and conclusions were re-examined using normal critical values, school-clustered CR1 standard errors, and models omitting either the age or the teaching experience controls (Table S5 in S1 File). The primary scales were fixed according to official OECD scoring before any significance results were inspected.

## 4. Results

### 4.1. Descriptive statistics and correlations

The primary analysis uses the four OECD TALIS scale scores: teacher-student relations (T4STUD, *X*), joy of teaching (T4JOYTCH, *M*), teacher job satisfaction (T4JOBSAT, *Y*), and principal relational leadership (T4RELEAD, *W*). The analytic sample comprises 1,338 teachers from 205 schools; the item-complete sample of *N* = 1,311 is used only for sensitivity analyses. Descriptive statistics and correlations, computed with the final teacher weight, are reported in Table 2. All four variables were positively intercorrelated. The correlation between joy of teaching and job satisfaction was the strongest (*r* = 0.599), whereas the correlations of teacher-student relations with job satisfaction (*r* = 0.453) and with joy of teaching (*r* = 0.452) were of similar magnitude.

### 4.2. Model 4: The statistical indirect association through joy of teaching

Regression models used the final teacher weight for point estimation and the 100 replicate weights with *ρ* = 0.5 for variance estimation, with inference based on the *t*(99) distribution. All models adjust for gender, age, educational attainment, and teaching experience, and the continuous focal variables were centered at their weighted means. The principal estimates are reported in Table 3.

**Table 3:** Survey-weighted Model 4 estimates.

Path or derived association	*B*	Fay-BRR *SE*	*t*(99)	*p*	95% CI
Total association, *X* to *Y* (*c*)	0.4371	0.0278	15.705	< 0.001	[0.3819, 0.4923]
*X* to *M* (*a*)	0.4631	0.0284	16.293	< 0.001	[0.4067, 0.5195]
*M* to *Y*, adjusted for *X* (*b*)	0.4603	0.0286	16.094	< 0.001	[0.4036, 0.5171]
Direct association, *X* to *Y*, adjusted for *M* (*c*′)	0.2239	0.0285	7.847	< 0.001	[0.1673, 0.2806]
Indirect association (*a* × *b*)	0.2132	0.0193	11.027	< 0.001	[0.1748, 0.2515]

*Note.* Unstandardized coefficients, adjusted for the four control variables.

The total association between teacher-student relations and job satisfaction was positive and significant (*c*: *B* = 0.4371, *SE* = 0.0278, *t* = 15.705, *p* < 0.001, 95% CI [0.3819, 0.4923]), supporting H1. Teacher-student relations were positively associated with joy of teaching (*a*: *B* = 0.4631, *SE* = 0.0284, *t* = 16.293, *p* < 0.001, 95% CI [0.4067, 0.5195]), suppor*t*ing H2. With teacher-student relations and the controls held constant, joy of teaching remained positively associated with job satisfaction (*b*: *B* = 0.4603, *SE* = 0.0286, *t* = 16.094, *p* < 0.001, 95% CI [0.4036, 0.5171]), supporting H3. Wi*t*h joy of teaching in the model, the direct association between teacher-student relations and job satisfaction remained positive (*c*′: *B* = 0.2239, *SE* = 0.0285, *t* = 7.847, *p* < 0.001, 95% CI [0.1673, 0.2806]).

The indirect association was estimated at 0.2132 (*SE* = 0.0193, *t* = 11.027, *p* < 0.001, 95% CI [0.1748, 0.2515]). Because this interval excludes zero, H4 is supported in the sense of a statistical association. The weighted R² was 0.21851 for the total association model with job satisfaction as the outcome, 0.21853 for the a path model with joy of teaching as the outcome, and 0.4058 for the outcome model containing both X and M.

### 4.3. Model 15: Tests of moderation by principal relational leadership

The outcome equation of Model 15 includes *X*, *M*, *W*, *X* × *W*, and *M* × *W* simultaneously. The weighted *R*² for the full model was 0.4772, and adding the two interaction terms yielded Δ*R*² = 0.0079. The joint test of the two interactions was significant, *F*(2, 99) = 6.184, *p* = 0.0029, but the two single-term tests diverged. Results are reported in Table 4.

**Table 4:** Survey-weighted Hayes Model 15 estimates.

Predictor or derived quantity	*B*	Fay-BRR *SE*	*t*(99)	*p*	95% CI
Conditional *X* to *Y* at weighted-mean *W*	0.0356	0.0297	1.201	0.2326	[-0.0232,0.0945]
Conditional *M* to *Y* at weighted-mean *W*	0.4021	0.0288	13.937	< 0.001	[0.3448, 0.4593]
Principal relational leadership (*W*, centered)	0.3560	0.0317	11.235	< 0.001	[0.2931, 0.4188]
*X* × *W*	−0.0057	0.0112	−0.512	0.6101	[-0.0280,0.0165]
*M* × *W*	0.0477	0.0143	3.341	0.0012	[0.0194,0.0760]
Index of moderated mediation (*a* × *b*_3_)	0.0221	0.0069	3.222	0.0017	[0.0085, 0.0357]

*Note. X*, *M*, and *W* are centered at their weighted means; control coefficients are omitted.

The interaction between teacher-student relations and principal relational leadership was not significant (*X* × *W*: *B* = −0.0057, *SE* = 0.0112, *t* = −0.512, *p* = 0.6101, 95% CI [−0.0280, 0.0165]). The data therefore do not support the hypothesis that *W* alters the conditional direct association between *X* and *Y*, and H5 is not supported.

The interaction between joy of teaching and principal relational leadership was positive and significant (*M* × *W*: *B* = 0.0477, *SE* = 0.0143, *t* = 3.341, *p* = 0.0012, 95% CI [0.0194, 0.0760]). In the official-scale primary analysis, the positive association between *M* and *Y* was stronger at higher levels of *W*, and H6 is therefore supported. The index of moderated mediation was 0.0221 (*SE* = 0.0069, *t* = 3.222, *p* = 0.0017, 95% CI [0.0085, 0.0357]), indicating that the conditional indirect association from *X* through *M* to *Y* increases with *W*.

**Fig 3:**
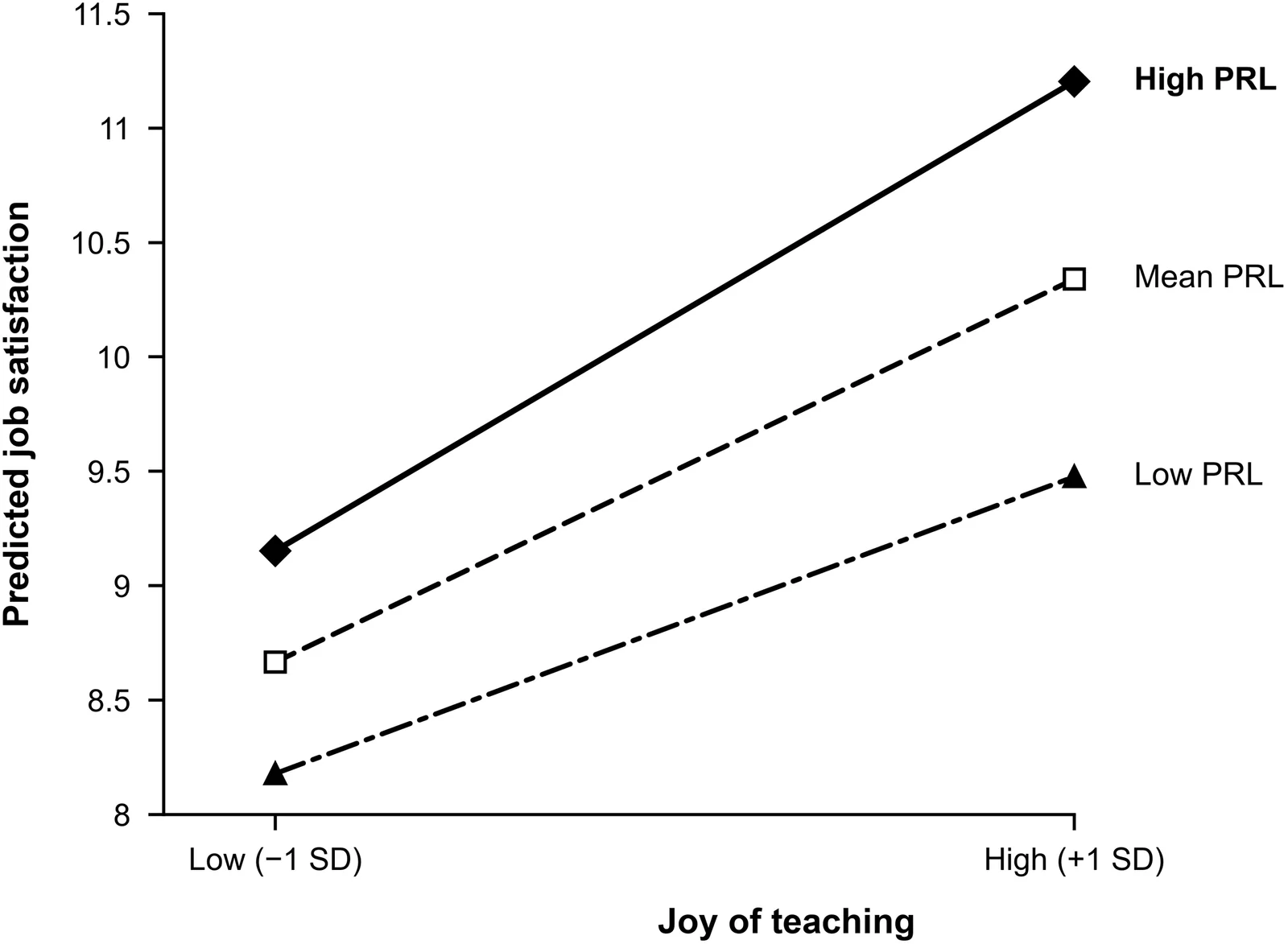
Predicted overall teacher job satisfaction by joy of teaching and teacher-perceived principal relational leadership (*N* = 1,338 teachers from 205 schools). Alt text: A two-point interaction plot shows predicted job satisfaction at low and high joy of teaching. All three lines slope upward, and the slope is steepest at high teacher-perceived principal relational leadership, intermediate at the mean, and shallowest at low leadership.

At one weighted standard deviation below the weighted mean of W, at the weighted mean, and at one weighted standard deviation above it, the conditional M to Y associations were 0.3116, 0.4021, and 0.4925, respectively, and the corresponding conditional indirect associations were 0.1443, 0.1862, and 0.2281; none of these intervals included zero. By contrast, the intervals for all three conditional direct associations between *X* and *Y* included zero, consistent with the nonsignificant *X* × *W* term (Table S6 in S1 File). To aid interpretation of the *M* × *W* interaction, Fig 3 displays predicted job satisfaction from the official-scale Model 15 at low and high levels of joy of teaching and of perceived principal relational leadership, each evaluated at its weighted mean and at plus or minus one weighted standard deviation with covariates held constant; the plotted lines represent conditional associations rather than causal effects.

### 4.4. Sensitivity to measurement operationalization

The official-scale analysis retains 27 more teachers than the item-complete sample used previously. To separate the effect of sample composition from that of scoring operationalization, the official scales were first restricted to the same *N* = 1,311: both *M* × *W* (*B* = 0.0465, *p* = 0.0021) and the index of moderated mediation (*B* = 0.0219, *p* = 0.0030) remained significant, indicating that the difference from the earlier results is not attributable to the 27 additional teachers (Table S5 in S1 File).

When *X*, *M*, *Y*, and *W* were all replaced by the simple item means used previously, *M* × *W* (*B* = 0.1009, *p* = 0.1282), the index of moderated mediation (*B* = 0.0484, *p* = 0.1339), and *X* × *W* (*B* = 0.0120, *p* = 0.7965) were all nonsignificant. H6 and the index therefore receive statistical support under both official-score specifications but not under the all-item-mean specification. A decomposition of the outcome shows that this divergence is associated primarily with the operationalization of job satisfaction: the interaction signal is concentrated in satisfaction with the work environment (*M* × *W*: *B* = 0.0549, *p* < 0.001) rather than satisfaction with the profession (*B* = 0.0262, *p* = 0.0691), and the paired standardized contrast between the two subscales was significant (*p* = 0.0273). The moderation result should accordingly be interpreted as exploratory and sensitive to measurement operationalization rather than as robust across operationalizations.

### 4.5. Sample selection and complex-sample robustness

Twenty-one Form A respondents did not enter the analytic sample: 4 lacked only official scale scores, 16 lacked only control variables, and 1 lacked both. Among the categories for which these respondents still provided valid answers, the weighted proportions of men, of teachers aged 50 or older, of teachers at ISCED level 6 or below, and of teachers with 11–20 years of experience were comparatively high. However, the group comprises only 21 respondents, with valid values on gender, age, educational attainment, and teaching experience for only 14, 19, 19, and 10 of them, so these differences are descriptive selection diagnostics rather than a basis for population inference (Table S2 in S1 File). Both the analytic sample of *N* = 1,338 and the item-complete sample of *N* = 1,311 cover all 205 schools, and the largest weighted compositional differences between them were 0.049, 0.788, 0.186, and 0.550 percentage points for the four control variables, indicating that the 27 additional teachers did not appreciably change the observed demographic composition.

Because school clustering is nonzero (Table S3 in S1 File), uncorrected independent-observation ordinary least squares results are reported only for comparison in Table S4 in S1 File and are not used for formal hypothesis decisions. When estimated with the final teacher weight and school-level CR1 standard errors, the *p* values for the official-scale *M* × *W* interaction and the index of moderated mediation were 0.0011 and 0.0012, consistent with the Fay-BRR values of 0.0012 and 0.0017; all key Model 4 paths remained significant and *X* × *W* remained nonsignificant. Uncorrected unweighted ordinary least squares produced the same pattern of significance, but its independent-observation standard errors do not reflect the complex TALIS design. Formal conclusions therefore rest on the weighted Fay-BRR results (Table S4 in S1 File), and a summary of all hypothesis decisions is provided in Table S7 in S1 File.

## 5. Discussion

### 5.1. Principal findings

Using design-based inference, this study examined the conditional associations among perceived school-wide teacher-student relations, joy of teaching, overall job satisfaction, and principal relational leadership for lower-secondary teachers in Shanghai. Perceptions of school-wide teacher-student relations were positively associated with both job satisfaction and joy of teaching; with those relations and the background variables held constant, joy of teaching remained significantly related to job satisfaction; and the Model 4 indirect association was positive with a confidence interval excluding zero. H1 through H4 are thus supported in the sense of statistical association, and joy of teaching emerges as an important emotional indicator for understanding the covariation between perceived relational climate and job satisfaction.

Model 15 produced divergent interaction results. Principal relational leadership did not significantly alter the conditional direct association between teacher-student relations and job satisfaction, so H5 is not supported; the interaction between joy of teaching and relational leadership was positive and the interval for the index of moderated mediation excluded zero, so H6 is supported in the primary analysis based on the official TALIS scales. That the two interaction terms differ in their significance decisions does not establish that the difference between them is itself significant. Sensitivity analyses bound this finding: restricting the official scales to the same 1,311 teachers left the conclusions unchanged, but the item-mean specification yielded nonsignificant estimates, and the interaction signal originated primarily in satisfaction with the work environment. H6 should therefore be regarded as exploratory and contingent on how the outcome is operationalized.

### 5.2. Theoretical implications

The results are consistent with the basic AET proposition that the work relational environment, positive emotion, and work attitudes are linked [22]: perceptions of school-wide teacher-student relations were positively related to joy of teaching, and joy of teaching was in turn related to overall job satisfaction. AET thus offers a useful framework for organizing these variables in educational settings.

The result for H6 provides preliminary evidence of context dependence in the linkage between emotion and work attitudes. Relational leadership emphasizes trust, communication, participation, and support [43]; where teachers perceive such practices more strongly, positive emotion during teaching may be more congruent with their overall appraisal of the work environment, an interpretation directionally consistent with research on leadership support and teacher well-being [18–21]. The idea that relational leadership functions as an affective amplifier nevertheless remains to be tested longitudinally.

The absence of support for H5 indicates that, once joy of teaching, perceived leadership, and the controls are in the model, the interval for *X* × *W* includes zero. Because the mediator equation contains no *X* × *W* term, this cannot be extended to first-stage moderation, nor does it show that the association between teacher-student relations and job satisfaction is independent of school leadership.

### 5.3. Practical implications

The results suggest that efforts to improve teachers’ occupational well-being may usefully attend simultaneously to the relational environment, teaching emotion, and leadership interaction. School administrators may provide a more consistent organizational backdrop for positive instructional experience by improving communication, widening teacher participation in important decisions, offering professional recognition, and providing timely support, and professional development may attend to classroom interaction, emotional awareness, and sustainable workload arrangements rather than locating responsibility for satisfaction solely with individual teachers.

Because no intervention was implemented, these suggestions are more appropriately used to identify relational leadership, joy of teaching, and satisfaction with the work environment as indicators worth monitoring jointly, with the effects of specific measures evaluated through school-level pilots, longitudinal follow-up, or quasi-experimental designs. Given that the signal for H6 originated primarily in satisfaction with the work environment, practical recommendations should give priority to teachers’ appraisals of their working conditions.

### 5.4. Limitations and future research

First, the study is cross-sectional and the focal variables were measured concurrently, so temporal ordering cannot be established, and both reverse and reciprocal associations remain possible. Teachers with higher job satisfaction may, for example, evaluate teacher-student relations, teaching experience, and principal leadership more favorably. Future work should use longitudinal designs with at least three waves that measure the relational environment, teaching emotion, and work attitudes separately, or apply cross-lagged, random-intercept cross-lagged, or natural experiment designs.

Second, all focal variables are teacher self-reports and may be affected by common method variance, general positive affect, and social desirability. Harman’s single-factor test cannot rule out such risks, and this study therefore does not interpret a first factor below an arbitrary threshold as evidence that common method bias is absent. Subsequent research could combine student reports, classroom observation, principal reports, and administrative data, and could separate data sources across measurement occasions.

Third, the study population comprises only ISCED 2 teachers in Shanghai (China) in TALIS 2024. Although the final teacher weight and replicate weights support design-based inference to the corresponding Shanghai target population, the results cannot be generalized automatically to other regions of China, other school levels, or different governance contexts. Cross-regional replication and tests of measurement invariance are necessary next steps.

Fourth, relational leadership is a teacher-level perceptual indicator. ICC(1) values ranged from 0.0571 to 0.0919, indicating nonzero within-school clustering; Fay-BRR and school-level CR1 agreed on all key significance decisions, but neither decomposes within-school and between-school associations into distinct parameters. The results therefore represent conditional associations at the level of teacher perception and cannot be read as cross-level effects of objective school leadership or of a school-average leadership climate. Because each school contributed only 4–9 teachers, and because an appropriate multilevel conditional process model would require level-specific weight scaling and within-between decomposition, no post hoc multilevel model with a different substantive meaning was added. Future work could test teacher-level and school-level interactions separately with larger within-school samples and use multilevel longitudinal designs [46].

Fifth, the moderation result is sensitive to scale operationalization. Official overall job satisfaction supports H6, whereas the previously used item means do not, and the official-scale result is driven primarily by the work environment subscale. The official scales rest on an explicit international calibration and are appropriate for the primary analysis, but the divergence indicates that construct boundaries and scoring choices affect inference. Future studies should specify the primary scales in advance, compare the two satisfaction subscales, and test the measurement model and latent interactions.

Sixth, questionnaire rotation restricts the joint model to Form A, and the official scales permit a score whenever at least two source items are answered. Only 21 Form A respondents were excluded, and missing controls partly define that group; their valid responses show comparatively high proportions of men, older teachers, teachers with lower educational attainment, and teachers with 11–20 years of experience, so selection bias cannot be entirely excluded. Restricting the official scales to the 1,311 item-complete respondents nevertheless left the conclusions unchanged, and the weighted composition of that subsample differs only marginally from the analytic sample. Because not all potential confounders, such as teacher self-efficacy, school resources, and workload, were measured, the adjusted associations should not be read as unbiased causal effects.

Finally, this study does not treat cross-sectional regressions with the variable order reversed as a test of causal direction. Fit indices for reversed models with different outcomes cannot show that one temporal ordering is more plausible, and exchanging *M* and *Y* does not resolve the absence of temporal precedence in concurrent self-report data. The study therefore reports only the statistical indirect association consistent with the theoretical pathway; direction and psychological mechanism require multi-source longitudinal data or experimental and quasi-experimental designs.

## 6. Conclusions

Using the official TALIS scales and design-based inference, this study finds that perceived school-wide teacher-student relations are positively associated with job satisfaction among lower-secondary teachers in Shanghai, and that joy of teaching constitutes a significant statistical indirect association between the two. Principal relational leadership did not significantly alter the conditional direct association between teacher-student relations and job satisfaction; in the official-scale primary analysis, however, higher perceived leadership was accompanied by a stronger positive association between joy of teaching and job satisfaction and by a stronger conditional indirect association. This moderation conclusion is sensitive to the operationalization of job satisfaction and is located primarily in satisfaction with the work environment. The results thus provide design-based evidence on the joint associations among relational environment, teaching emotion, and perceived leadership, while mechanisms, directionality, and school-level implications require multi-source, longitudinal, and multilevel research.

## Supporting information

S1 File Supporting information tables S1-S7. (DOCX)

S2 File Reproducibility materials, analysis script, and analysis outputs. (DOCX)
