# Supplementary material for: Perceived school-wide teacher-student relations and teacher job satisfaction: An affective events theory analysis of Shanghai teachers
Source: PLoS One. 2026 Sep 3;21(9):e0357279. doi: 10.1371/journal.pone.0357279 (PMC13541260; doi:10.1371/journal.pone.0357279)
Supplement: Supplementary file 1 [file pone.0357279.s001.docx]

# S1 File. Supporting information tables S1-S7

**Perceived School-Wide Teacher-Student Relations and Teacher Job Satisfaction: An Affective Events Theory Analysis of Shanghai Teachers**

**Abbreviations:** TALIS, Teaching and Learning International Survey; OECD, Organisation for Economic Co-operation and Development; ISCED, International Standard Classification of Education; BRR, balanced repeated replication; ICC, intraclass correlation coefficient; CR1, cluster-robust variance estimator with the standard finite-sample adjustment.

Unless otherwise indicated, all tables are based on *N* = 1,338 teachers from 205 schools and the official TALIS scales, with identical covariate coding; point estimates use the OECD final teacher weight, and design-based standard errors, confidence intervals, and *p* values use 100 replicate weights with Fay’s *ρ* = 0.5 and *t*(99) inference. The derivation sequence was 4,078 teachers, then Form A (*n* = 1,359), then all four official scores available (*n* = 1,354), then complete controls (*n* = 1,338); Forms B and C (*n* = 2,719) were structurally ineligible. No cases were excluded as outliers.

**Table S1. Questionnaire rotation, marginal missingness, and derivation of the analytic sample.**

| **Variable** | **Description** | **Administered forms** | **Planned not administered, full file *n*** | **Ordinary missing, full file *n*** | **Ordinary missing, Form A *n*** | **Valid Form A *n*** |
| --- | --- | --- | --- | --- | --- | --- |
| T4STUD | Teacher-student relations | A and C | 1,355 | 8 | 4 | 1,355 |
| T4JOYTCH | Joy of teaching | A and B | 1,364 | 5 | 3 | 1,356 |
| T4JOBSAT | Job satisfaction, overall | A, B, and C | 0 | 9 | 3 | 1,356 |
| T4RELEAD | Relational leadership | A and C | 1,355 | 8 | 3 | 1,356 |
| TT4G01 | Gender | A, B, and C | 0 | 22 | 7 | 1,352 |
| T4TAGEGR | Age group | A, B, and C | 0 | 9 | 2 | 1,357 |
| T4THEDAT | Highest educational attainment | A and B | 1,364 | 4 | 2 | 1,357 |
| T4TYEXPTT | Total teaching experience | A, B, and C | 0 | 44 | 11 | 1,348 |

*Note.* Marginal missing counts overlap and must not be summed. Of the 21 Form A respondents not included, 4 lacked only official scores, 16 lacked only controls, and 1 lacked both.

**Table S2. Survey-weighted comparison of included and not-included Form A respondents.**

| **Characteristic** | **Category** | **Included *n*** | **Included weighted %** | **Not included *n*** | **Not included weighted %** | **Difference, percentage points** |
| --- | --- | --- | --- | --- | --- | --- |
| Gender | Female | 984 | 74.19 | 6 | 43.26 | +30.94 |
| Gender | Male | 354 | 25.81 | 8 | 56.74 | -30.94 |
| Age group | Under 30 years | 249 | 19.61 | 3 | 19.17 | +0.44 |
| Age group | 30 to 39 years | 413 | 30.82 | 1 | 5.52 | +25.30 |
| Age group | 40 to 49 years | 400 | 29.52 | 7 | 37.54 | -8.02 |
| Age group | 50 years or older | 276 | 20.05 | 8 | 37.77 | -17.72 |
| Education | ISCED 6 or below | 1,038 | 77.18 | 18 | 94.56 | -17.38 |
| Education | ISCED 7 or 8 | 300 | 22.82 | 1 | 5.44 | +17.38 |
| Teaching experience | 5 years or less | 269 | 21.17 | 1 | 13.44 | +7.73 |
| Teaching experience | 6 to 10 years | 243 | 17.81 | 2 | 20.12 | -2.31 |
| Teaching experience | 11 to 20 years | 317 | 23.46 | 5 | 47.23 | -23.78 |
| Teaching experience | More than 20 years | 509 | 37.57 | 2 | 19.21 | +18.36 |

*Note.* Percentages use nonmissing responses within each group; difference = included minus not included. Weighted Cramér’s *V* was 0.070, 0.071, 0.048, and 0.051 for the four characteristics, and comparisons of Form A with Forms B and C gave values of 0.011 to 0.030 with category differences of 2.14 percentage points or less. Because the comparison group is very small and missing controls partly define exclusion, these are descriptive diagnostics, not design-based tests.

**Table S3. School cluster sizes, intraclass correlations, and approximate design effects.**

| **Official scale** | **Teachers, *n*** | **Schools, *n*** | **ICC(1)** | **Effective mean cluster size** | **Approximate design effect** |
| --- | --- | --- | --- | --- | --- |
| Teacher-student relations (T4STUD) | 1,338 | 205 | 0.0571 | 6.526 | 1.316 |
| Joy of teaching (T4JOYTCH) | 1,338 | 205 | 0.0738 | 6.526 | 1.408 |
| Overall job satisfaction (T4JOBSAT) | 1,338 | 205 | 0.0890 | 6.526 | 1.492 |
| Principal relational leadership (T4RELEAD) | 1,338 | 205 | 0.0919 | 6.526 | 1.508 |

*Note.* ICCs are unweighted one-way random-intercept diagnostics. Cluster sizes were 4 to 9 teachers per school (*Q*1 = 6, median = 7, arithmetic mean = 6.527, *Q*3 = 7), with no singleton schools.

**Table S4. Conventional, school-cluster-robust, and design-based inference compared.**

| Parameter | Unweighted ordinary least squares: *B* (*SE*), 95% CI; *p* | Weighted with school CR1: *B* (*SE*), 95% CI; *p* | Weighted with Fay-BRR: *B* (*SE*), 95% CI; *p* | Decision |
| --- | --- | --- | --- | --- |
| *a* | 0.4704 (0.0249), [0.4215, 0.5193];  *p* < 0.001 | 0.4631 (0.0271), [0.4096, 0.5166];  *p* < 0.001 | 0.4631 (0.0284), [0.4067, 0.5195];  *p* < 0.001 | Significant in all |
| *b* | 0.4676 (0.0225), [0.4234, 0.5118];  *p* < 0.001 | 0.4603 (0.0275), [0.4062, 0.5144];  *p* < 0.001 | 0.4603 (0.0286), [0.4036, 0.5171];  *p* < 0.001 | Significant in all |
| *c* | 0.4458 (0.0236), [0.3996, 0.4920];  *p* < 0.001 | 0.4371 (0.0264), [0.3850, 0.4892];  *p* < 0.001 | 0.4371 (0.0278), [0.3819, 0.4923];  *p* < 0.001 | Significant in all |
| *c*′ | 0.2259 (0.0231), [0.1806, 0.2711];  *p* < 0.001 | 0.2239 (0.0285), [0.1678, 0.2801];  *p* < 0.001 | 0.2239 (0.0285), [0.1673, 0.2806];  *p* < 0.001 | Significant in all |
| *a* × *b* | 0.2200 (0.0158), [0.1890, 0.2509];  *p* < 0.001 | 0.2132 (0.0187), [0.1763, 0.2501];  *p* < 0.001 | 0.2132 (0.0193), [0.1748, 0.2515];  *p* < 0.001 | Significant in all |
| *X* × *W* | -0.0095 (0.0111),  [-0.0314, 0.0123];  *p* = 0.3915 | -0.0057 (0.0114),  [-0.0282, 0.0167];  *p* = 0.6157 | -0.0057 (0.0112),  [-0.0280, 0.0165];  *p* = 0.6101 | Nonsignificant in all |
| *M* × *W* | 0.0543 (0.0110), [0.0328, 0.0758];  *p* < 0.001 | 0.0477 (0.0144), [0.0194, 0.0760];  *p* = 0.0011 | 0.0477 (0.0143), [0.0194, 0.0760];  *p* = 0.0012 | Significant in all |
| *a* × *b*_3_ | 0.0256 (0.0053), [0.0151, 0.0360];  *p* < 0.001 | 0.0221 (0.0067), [0.0088, 0.0354];  *p* = 0.0012 | 0.0221 (0.0069), [0.0085, 0.0357];  *p* = 0.0017 | Significant in all |

*Note.* Ordinary least squares uses model-based independent-observation standard errors; *a* × *b* and *a* × *b*_3_ use a joint first-order delta method retaining cross-equation residual covariance. School CR1 uses *df* = 204. CR1 and Fay-BRR standard errors were not combined.

**Table S5. Measurement and outcome-operationalization sensitivity analyses.**

| **Specification** | ***X* × *W*: *B* (*SE*), 95% CI; *p*** | ***M* × *W*: *B* (*SE*), 95% CI; *p*** | **Index of moderated mediation: *B* (*SE*), 95% CI; *p*** |
| --- | --- | --- | --- |
| *Panel A. Whole-construct operationalization* |  |  |  |
| Official TALIS scores, maximum available sample (*N* = 1,338) | -0.0057 (0.0112),  [-0.0280, 0.0165];  *p* = 0.6101 | 0.0477 (0.0143), [0.0194, 0.0760]; *p* = 0.0012 | 0.0221 (0.0069), [0.0085, 0.0357]; *p* = 0.0017 |
| Official TALIS scores, common sample (*N* = 1,311) | -0.0053 (0.0114),  [-0.0279, 0.0172];  *p* = 0.6390 | 0.0465 (0.0147), [0.0173, 0.0758]; *p* = 0.0021 | 0.0219 (0.0072), [0.0076, 0.0361]; *p* = 0.0030 |
| Raw item means, common sample (*N* = 1,311) | 0.0120 (0.0466),  [-0.0803, 0.1044];  *p* = 0.7965 | 0.1009 (0.0657),  [-0.0296, 0.2313]; *p* = 0.1282 | 0.0484 (0.0320),  [-0.0152, 0.1120]; *p* = 0.1339 |
| *Panel B. Outcome operationalization with official X, M, and W fixed (N = 1,311)* |  |  |  |
| T4JOBSAT, overall job satisfaction | Not applicable | 0.0465 (0.0147), [0.0173, 0.0758]; *p* = 0.0021 | 0.0219 (0.0072), [0.0076, 0.0361];  *p* = 0.0030 |
| T4JSPROT, satisfaction with the profession | Not applicable | 0.0262 (0.0143),  [-0.0021, 0.0545]; *p* = 0.0691 | 0.0123 (0.0069),  [-0.0013, 0.0260];  *p* = 0.0763 |
| T4JSENVT, satisfaction with the work environment | Not applicable | 0.0549 (0.0141), [0.0270, 0.0828]; *p* < 0.001 | 0.0258 (0.0068), [0.0123, 0.0393];  *p* < 0.001 |
| Six-item outcome mean used previously | Not applicable | 0.0082 (0.0047),  [-0.0012, 0.0175]; *p* = 0.0870 | 0.0038 (0.0022),  [-0.0006, 0.0083];  *p* = 0.0898 |
| Paired standardized contrast: profession minus work environment | Not applicable | -0.0592,  [-0.1117, -0.0068]; *p* = 0.0273 | -0.0278, [-0.0523, -0.0033]; *p* = 0.0265 |

*Note.* Coefficient magnitudes are not directly comparable across differently scaled specifications. School-clustered CR1 checks gave *p* = 0.0011 and 0.0012 for the official-score *M* × *W* and index, and *p* = 0.1165 and 0.1190 for the corresponding item-mean quantities. Normal-theory critical values, and omitting the age or teaching experience controls, produced identical decisions.

**Table S6. Conditional associations at levels of principal relational leadership.**

| Level of *W* | *W* on official-score scale | Conditional *X* to *Y*: *B* (*SE*), 95% CI; *p* | Conditional *M* to *Y*: *B* (*SE*), 95% CI; *p* | Conditional indirect association: *B* (*SE*), 95% CI; *p* |
| --- | --- | --- | --- | --- |
| Low (*M* - 1 *SD*) | 11.341 | 0.0465 (0.0287),  [-0.0104, 0.1034];  *p* = 0.1083 | 0.3116 (0.0382), [0.2357, 0.3874];  *p* < 0.001 | 0.1443 (0.0194), [0.1058, 0.1828];  *p* < 0.001 |
| Mean | 13.238 | 0.0356 (0.0297),  [-0.0232, 0.0945];  *p* = 0.2326 | 0.4021 (0.0288), [0.3448, 0.4593];  *p* < 0.001 | 0.1862 (0.0174), [0.1517, 0.2207];  *p* < 0.001 |
| High (*M* + 1 *SD*) | 15.135 | 0.0248 (0.0429),  [-0.0604, 0.1099];  *p* = 0.5652 | 0.4925 (0.0409), [0.4114, 0.5736];  *p* < 0.001 | 0.2281 (0.0238), [0.1809, 0.2753];  *p* < 0.001 |

*Note.* Significance at each probed value of *W* does not replace the test of the index of moderated mediation.

**Table S7. Summary of hypothesis tests.**

| **Hypothesis** | **Statistical test** | **Decision** |
| --- | --- | --- |
| H1: *X* is positively associated with *Y*. | *c* = 0.4371, *p* < 0.001 | Supported |
| H2: *X* is positively associated with *M*. | *a* = 0.4631, *p* < 0.001 | Supported |
| H3: *M* is positively associated with *Y*. | *b* = 0.4603, *p* < 0.001 | Supported |
| H4: *X* has an indirect association with *Y* through *M*. | *a* × *b* = 0.2132, 95% CI [0.1748, 0.2515] | Supported as a statistical indirect association |
| H5: *W* moderates the direct *X* to *Y* association. | *X* × *W* = -0.0057, *p* = 0.6101 | Not supported |
| H6: *W* moderates the *M* to *Y* association and the conditional indirect association. | *M* × *W* = 0.0477, *p* = 0.0012; index = 0.0221, *p* = 0.0017 | Supported in the declared official-score primary model, but measurement-sensitive and exploratory; evidence concentrated in work-environment satisfaction |

*Note.* H5 tests *X* × *W* in the outcome equation, not first-stage moderation. All decisions are associational because the data are cross-sectional.
