# Supplementary material for: Perceived school-wide teacher-student relations and teacher job satisfaction: An affective events theory analysis of Shanghai teachers
Source: PLoS One. 2026 Sep 3;21(9):e0357279. doi: 10.1371/journal.pone.0357279 (PMC13541260; doi:10.1371/journal.pone.0357279)
Supplement: Supplementary file 2 [file pone.0357279.s002.docx]

**S2 File**

Reproducibility materials, analysis script, and analysis outputs

*Perceived School-Wide Teacher-Student Relations and Teacher Job Satisfaction: An Affective Events Theory Analysis of Shanghai Teachers*

**Rerun status:** Passed. All 18 core benchmark estimates matched the manuscript at the reported precision; the corrected descriptive alpha and two paired Table S5 contrasts also matched the manuscript and S1 File. No respondent-level data are included.

# **1. Purpose and scope**

This document is the complete reproducibility record for the article named above. It is self-contained: the analysis specification, the full analysis script, the variable mapping, the key aggregate outputs, the software environment, and the integrity checks are reproduced here as text and tables, with no companion archive required.

The package reproduces the analytic sample derivation, the descriptive statistics, Model 4, Model 15, Fay balanced repeated replication (BRR) inference, and the sensitivity analyses reported in Tables S1-S7 of the S1 File.

The independently verified corrected run was performed from the original OECD TALIS 2024 Shanghai teacher public-use file on 18 August 2026. The source file was read but never modified; the script writes only aggregate tables, diagnostics, and one figure.

# **2. Input data and integrity**

The analysis requires the TALIS 2024 teacher public-use file for Shanghai (China). It is not redistributed with this document.

| Field | Validated value |
| --- | --- |
| Expected file | TALIS2024_BTG_CSH_teacher.sav |
| SHA-256 of input | b4f63148a6eba46ef8f88619b834f660414767cc3f860857faa156953356b7fc |
| Full file | 4,078 teachers; 205 schools |
| Eligible questionnaire form | Form A; n = 1,359 |
| Primary analytic sample | 1,338 teachers; 205 schools |
| Complete-item sensitivity sample | 1,311 teachers; 205 schools |

The file is available from the OECD TALIS 2024 Database at https://www.oecd.org/en/data/datasets/talis-2024-database.html. Access is administered solely by the OECD through its Data Access and Research Information Survey: users provide institutional and contact information, describe their research project, and agree to the OECD Terms of Use before downloading. No author-mediated approval is involved, and the authors have no role in granting or facilitating access. Questions about access may be directed to the OECD TALIS Secretariat at edutaliscontact@oecd.org.

# **3. How to reproduce**

Appendix A contains the complete analysis script. To rerun the analysis:

(i) Copy the text of Appendix A into a plain-text editor and save it as S2_analysis.R using UTF-8 encoding. Do not save it from a word processor, which may substitute typographic quotation marks and dashes for the ASCII characters the script requires.

(ii) Obtain TALIS2024_BTG_CSH_teacher.sav from the OECD as described in Section 2 and verify its SHA-256 against the value in the table above.

(iii) To avoid locale-dependent path errors, place S2_analysis.R, TALIS2024_BTG_CSH_teacher.sav, and the output directory in a path containing only ASCII characters (for example, C:/TALIS2024/).

(iv) From a terminal with Rscript on the system path, run the command below.

Rscript S2_analysis.R "path/to/TALIS2024_BTG_CSH_teacher.sav" "path/to/output_directory"

The script writes the complete set of tables and figures used for the manuscript and supporting analyses, together with a run log and the integrity checks in Section 5. Appendix C displays the key outputs, and the validated environment is reported in Appendix D.

# **4. Analysis specification**

The primary analysis uses the OECD scales T4STUD (X), T4JOYTCH (M), T4JOBSAT (Y), and T4RELEAD (W). Gender, age, education, and total teaching experience are included as predetermined controls. X, M, and W are centered at their final-teacher-weighted means.

Point estimates use the final teacher weight TCHWGT. Sampling variance is estimated from the 100 teacher replicate weights TRWGT1 to TRWGT100 using Fay BRR with the Fay coefficient set to rho = 0.5, and inference uses the t(99) distribution. Products and conditional associations are recomputed within every replicate before their design-based variance is estimated.

The raw-item sensitivity outcome uses TT4G78A, TT4G78B, TT4G78E, TT4G78G, TT4G78H, and TT4G78J. This six-item definition recovers both the reported N = 1,311 sample and the published sensitivity estimates.

Weighted cluster-robust (CR1) standard errors across the 205 schools are computed as a sensitivity check only and are not combined with the Fay-BRR standard errors.

# **5. Integrity checks**

| Check | Observed | Expected | Status |
| --- | --- | --- | --- |
| Rows | 4078 | 4078 | PASS |
| Form A respondents | 1359 | 1359 | PASS |
| Analytic sample | 1338 | 1338 | PASS |
| Item-complete sample | 1311 | 1311 | PASS |
| Schools in analytic sample | 205 | 205 | PASS |
| Reported estimates matched at printed precision | 18 | 18 | PASS |

Implementation verification: user-missing codes are removed before reverse scoring, and the paired Table S5 contrast is reported in the weighted-SD metric. These checks reproduce alpha = 0.845018 and the two S1 File contrasts without changing the 18 core benchmarks.

# **6. Key reproduced estimates**

| Quantity | B | Fay-BRR SE | p |
| --- | --- | --- | --- |
| Total association c | 0.4371 | 0.0278 | < 0.001 |
| X to M path a | 0.4631 | 0.0284 | < 0.001 |
| M to Y path b | 0.4603 | 0.0286 | < 0.001 |
| Direct association c' | 0.2239 | 0.0285 | < 0.001 |
| Indirect association a x b | 0.2132 | 0.0193 | < 0.001 |
| X x W | -0.0057 | 0.0112 | 0.6101 |
| M x W | 0.0477 | 0.0143 | 0.0012 |
| Index of moderated mediation a x b3 | 0.0221 | 0.0069 | 0.0017 |

Appendix C reports the key aggregate outputs, including the observed-versus-expected comparison against every benchmark estimate printed in the manuscript and the verified implementation details.

# **7. Contents of this document**

| Section | Content |
| --- | --- |
| Sections 1-6 | Scope, input identity, reproduction instructions, analysis specification, integrity checks, key estimates |
| Section 8 | Interpretation boundary |
| Appendix A | Complete corrected analysis script (S2_analysis.R), 1,298 lines |
| Appendix B | Variable mapping: source variables, analytic role, source labels, and missing-value codes |
| Appendix C | Key aggregate outputs: sample flow, demographics, descriptives and correlations, Model 4, Model 15, model fit, joint Wald test, figure plot data, validation against the manuscript, and verified implementation details |
| Appendix D | Software session information and input-file identity |

Tables S1-S7 are presented in the S1 File and are not duplicated here; the script in Appendix A regenerates all of them.

# **8. Interpretation boundary**

The rerun confirms computational consistency, not causal identification. The cross-sectional ordering cannot establish temporal direction, the models estimate teacher-level associations, and the results do not identify school-level leadership effects.

H1-H4 are reproduced as statistical associations. H5 is not supported. H6 is supported under the declared official-score primary model but not under the all-item-mean specification, so it remains exploratory and measurement-sensitive.

# **Appendix A. Analysis script**

The following is the complete text of S2_analysis.R. It is reproduced verbatim in a fixed-width font. See Section 3 for saving and running instructions.

```
#!/usr/bin/env Rscript

# Reproducibility analysis for:
# Perceived School-Wide Teacher-Student Relations and Teacher Job Satisfaction:
# An Affective Events Theory Analysis of Shanghai Teachers
#
# Usage:
#   Rscript S2_analysis.R path/to/TALIS2024_BTG_CSH_teacher.sav output_directory
#
# The original OECD public-use file is read but never modified. Only aggregate
# tables, diagnostics, and a figure are written to output_directory.

suppressPackageStartupMessages({
  library(haven)
  library(survey)
  library(sandwich)
  library(psych)
  library(ggplot2)
  library(dplyr)
  library(tidyr)
  library(readr)
})

args <- commandArgs(trailingOnly = TRUE)
if (length(args) != 2L) {
  stop("Usage: Rscript S2_analysis.R INPUT_SAV OUTPUT_DIRECTORY")
}

input_path <- normalizePath(args[[1]], mustWork = TRUE, winslash = "/")
output_dir <- normalizePath(args[[2]], mustWork = FALSE, winslash = "/")
dir.create(output_dir, recursive = TRUE, showWarnings = FALSE)

options(stringsAsFactors = FALSE, scipen = 999)

write_log <- function(...) {
  line <- paste0(format(Sys.time(), "%Y-%m-%d %H:%M:%S"), " | ", paste(..., collapse = ""))
  cat(line, "\n")
  cat(line, "\n", file = file.path(output_dir, "run_log.txt"), append = TRUE)
}

write_log("Starting analysis")
write_log("Input: ", input_path)
write_log("Output: ", output_dir)

required_versions <- c(
  haven = "2.5.5",
  survey = "4.5",
  ggplot2 = "4.0.3"
)
installed_versions <- vapply(
  names(required_versions),
  function(pkg) as.character(packageVersion(pkg)),
  character(1)
)
for (pkg in names(required_versions)) {
  if (installed_versions[[pkg]] != required_versions[[pkg]]) {
    warning(
      pkg, " version is ", installed_versions[[pkg]],
      "; manuscript reports ", required_versions[[pkg]]
    )
  }
}

# Read SPSS user-missing values as labelled values so planned non-administration
# (8/9998) can be distinguished from ordinary missingness (9/9999).
raw <- read_sav(input_path, user_na = TRUE)

raw_numeric <- function(x) {
  # Keep SPSS user-missing codes (8/9 and 9998/9999) intact for Table S1.
  as.numeric(x)
}

analysis_numeric <- function(x) {
  as.numeric(zap_labels(zap_missing(x)))
}

needed <- c(
  "IDTQUEST", "IDSCHOOL", "IDTEACH",
  "TCHWGT", paste0("TRWGT", 1:100),
  "T4STUD", "T4JOYTCH", "T4JOBSAT", "T4JSPROT", "T4JSENVT", "T4RELEAD",
  "TT4G01", "T4TAGEGR", "T4THEDAT", "T4TYEXPTT",
  paste0("TT4G65", LETTERS[1:4]),
  paste0("TT4G80", LETTERS[1:4]),
  paste0("TT4G66", LETTERS[5:8]),
  paste0("TT4G78", c("A", "B", "C", "D", "E", "G", "H", "J"))
)
missing_vars <- setdiff(needed, names(raw))
if (length(missing_vars)) {
  stop("Required variables missing: ", paste(missing_vars, collapse = ", "))
}

dat <- as.data.frame(lapply(raw[needed], analysis_numeric))
raw_codes <- as.data.frame(lapply(raw[needed], raw_numeric))

stopifnot(
  nrow(dat) == 4078L,
  sum(raw_codes$IDTQUEST == 1, na.rm = TRUE) == 1359L,
  sum(raw_codes$IDTQUEST == 2, na.rm = TRUE) == 1355L,
  sum(raw_codes$IDTQUEST == 3, na.rm = TRUE) == 1364L
)

scale_vars <- c("T4STUD", "T4JOYTCH", "T4JOBSAT", "T4RELEAD")
control_vars <- c("TT4G01", "T4TAGEGR", "T4THEDAT", "T4TYEXPTT")
repweight_vars <- paste0("TRWGT", 1:100)

form_a <- dat$IDTQUEST == 1
scale_complete <- form_a & complete.cases(dat[scale_vars])
analytic_flag <- form_a & complete.cases(dat[c(scale_vars, control_vars)])

if (sum(scale_complete) != 1354L || sum(analytic_flag) != 1338L) {
  stop(
    "Sample derivation mismatch: scale-complete=", sum(scale_complete),
    ", analytic=", sum(analytic_flag)
  )
}

analysis <- dat[analytic_flag, , drop = FALSE]
analysis$male <- as.integer(analysis$TT4G01 == 2)
analysis$age30_39 <- as.integer(analysis$T4TAGEGR == 2)
analysis$age40_49 <- as.integer(analysis$T4TAGEGR == 3)
analysis$age50plus <- as.integer(analysis$T4TAGEGR %in% c(4, 5))
analysis$edu78 <- as.integer(analysis$T4THEDAT %in% c(4, 5))
analysis$exp6_10 <- as.integer(analysis$T4TYEXPTT == 2)
analysis$exp11_20 <- as.integer(analysis$T4TYEXPTT == 3)
analysis$exp20plus <- as.integer(analysis$T4TYEXPTT == 4)

control_terms <- c(
  "male", "age30_39", "age40_49", "age50plus",
  "edu78", "exp6_10", "exp11_20", "exp20plus"
)

wmean <- function(x, w) {
  ok <- is.finite(x) & is.finite(w)
  sum(w[ok] * x[ok]) / sum(w[ok])
}

wvar_pop <- function(x, w) {
  ok <- is.finite(x) & is.finite(w)
  m <- wmean(x[ok], w[ok])
  sum(w[ok] * (x[ok] - m)^2) / sum(w[ok])
}

wsd_pop <- function(x, w) sqrt(wvar_pop(x, w))

wcov_pop <- function(x, y, w) {
  ok <- is.finite(x) & is.finite(y) & is.finite(w)
  mx <- wmean(x[ok], w[ok])
  my <- wmean(y[ok], w[ok])
  sum(w[ok] * (x[ok] - mx) * (y[ok] - my)) / sum(w[ok])
}

wcor <- function(x, y, w) {
  wcov_pop(x, y, w) / sqrt(wvar_pop(x, w) * wvar_pop(y, w))
}

weighted_alpha <- function(items, w) {
  ok <- complete.cases(items) & is.finite(w)
  x <- as.matrix(items[ok, , drop = FALSE])
  ww <- w[ok]
  means <- colSums(x * ww) / sum(ww)
  centered <- sweep(x, 2, means, "-")
  cov_mat <- crossprod(centered * sqrt(ww), centered * sqrt(ww)) / sum(ww)
  k <- ncol(x)
  k / (k - 1) * (1 - sum(diag(cov_mat)) / sum(cov_mat))
}

center_constants <- c(
  X = wmean(analysis$T4STUD, analysis$TCHWGT),
  M = wmean(analysis$T4JOYTCH, analysis$TCHWGT),
  W = wmean(analysis$T4RELEAD, analysis$TCHWGT)
)
analysis$Xc <- analysis$T4STUD - center_constants[["X"]]
analysis$Mc <- analysis$T4JOYTCH - center_constants[["M"]]
analysis$Wc <- analysis$T4RELEAD - center_constants[["W"]]

w_sd <- wsd_pop(analysis$T4RELEAD, analysis$TCHWGT)
probe_centered <- c(low = -w_sd, mean = 0, high = w_sd)
probe_original <- center_constants[["W"]] + probe_centered

# Construct a formal survey design object as a structural check. Nonlinear
# statistics below are re-estimated with the same full and replicate weights.
rep_design <- svrepdesign(
  weights = ~TCHWGT,
  repweights = analysis[repweight_vars],
  data = analysis,
  type = "Fay",
  rho = 0.5,
  combined.weights = TRUE
)
if (survey::degf(rep_design) != 99L) {
  stop("Unexpected replicate-design degrees of freedom: ", survey::degf(rep_design))
}

weighted_lm <- function(formula, data, weights) {
  lm(formula, data = data, weights = weights, model = TRUE, x = TRUE, y = TRUE)
}

coef_named <- function(fit, term) {
  out <- coef(fit)[[term]]
  if (is.null(out) || !is.finite(out)) {
    stop("Coefficient not estimable: ", term)
  }
  unname(out)
}

brr_bundle <- function(data, estimator, label) {
  point <- estimator(data$TCHWGT, data)
  if (is.null(names(point)) || anyDuplicated(names(point))) {
    stop("Estimator must return uniquely named statistics: ", label)
  }
  reps <- t(vapply(
    repweight_vars,
    function(wname) estimator(data[[wname]], data),
    FUN.VALUE = point
  ))
  diffs <- sweep(reps, 2, point, "-")
  covariance <- (1 / (100 * (1 - 0.5)^2)) * crossprod(diffs)
  se <- sqrt(diag(covariance))
  t_value <- point / se
  p_value <- 2 * pt(-abs(t_value), df = 99)
  critical <- qt(0.975, df = 99)
  summary <- data.frame(
    metric = names(point),
    estimate = unname(point),
    se = unname(se),
    t = unname(t_value),
    df = 99,
    p = unname(p_value),
    ci_low = unname(point - critical * se),
    ci_high = unname(point + critical * se),
    stringsAsFactors = FALSE
  )
  list(
    label = label,
    point = point,
    replicates = reps,
    covariance = covariance,
    summary = summary
  )
}

model4_estimator <- function(weights, d) {
  controls <- paste(control_terms, collapse = " + ")
  total_fit <- weighted_lm(
    as.formula(paste("T4JOBSAT ~ Xc +", controls)), d, weights
  )
  mediator_fit <- weighted_lm(
    as.formula(paste("T4JOYTCH ~ Xc +", controls)), d, weights
  )
  outcome_fit <- weighted_lm(
    as.formula(paste("T4JOBSAT ~ Xc + Mc +", controls)), d, weights
  )
  c_path <- coef_named(total_fit, "Xc")
  a_path <- coef_named(mediator_fit, "Xc")
  b_path <- coef_named(outcome_fit, "Mc")
  c_prime <- coef_named(outcome_fit, "Xc")
  c(
    total_c = c_path,
    a = a_path,
    b = b_path,
    direct_cprime = c_prime,
    indirect_ab = a_path * b_path
  )
}

model15_estimator <- function(weights, d) {
  controls <- paste(control_terms, collapse = " + ")
  mediator_fit <- weighted_lm(
    as.formula(paste("T4JOYTCH ~ Xc +", controls)), d, weights
  )
  outcome_fit <- weighted_lm(
    as.formula(
      paste(
        "T4JOBSAT ~ Xc + Mc + Wc + Xc:Wc + Mc:Wc +",
        controls
      )
    ),
    d,
    weights
  )
  a_path <- coef_named(mediator_fit, "Xc")
  c_prime <- coef_named(outcome_fit, "Xc")
  b_path <- coef_named(outcome_fit, "Mc")
  w_main <- coef_named(outcome_fit, "Wc")
  c3 <- coef_named(outcome_fit, "Xc:Wc")
  b3 <- coef_named(outcome_fit, "Mc:Wc")
  out <- c(
    direct_at_mean_W = c_prime,
    M_to_Y_at_mean_W = b_path,
    W_main = w_main,
    X_by_W = c3,
    M_by_W = b3,
    index_a_b3 = a_path * b3
  )
  for (level in names(probe_centered)) {
    z <- probe_centered[[level]]
    out[[paste0("direct_", level)]] <- c_prime + c3 * z
    out[[paste0("M_to_Y_", level)]] <- b_path + b3 * z
    out[[paste0("indirect_", level)]] <- a_path * (b_path + b3 * z)
  }
  out
}

write_log("Running Model 4 with 100 Fay-BRR replicates")
model4_brr <- brr_bundle(analysis, model4_estimator, "Model 4")
write_log("Running Model 15 with 100 Fay-BRR replicates")
model15_brr <- brr_bundle(analysis, model15_estimator, "Model 15")

write_csv(model4_brr$summary, file.path(output_dir, "table_3_model4.csv"))

table4_metrics <- c(
  "direct_at_mean_W", "M_to_Y_at_mean_W", "W_main",
  "X_by_W", "M_by_W", "index_a_b3"
)
write_csv(
  model15_brr$summary |>
    filter(metric %in% table4_metrics),
  file.path(output_dir, "table_4_model15.csv")
)

table_s6 <- model15_brr$summary |>
  filter(grepl("^(direct|M_to_Y|indirect)_(low|mean|high)$", metric)) |>
  mutate(
    quantity = sub("_(low|mean|high)$", "", metric),
    level = sub("^.*_(low|mean|high)$", "\\1", metric),
    W_original_scale = unname(probe_original[level])
  ) |>
  select(level, W_original_scale, quantity, estimate, se, t, df, p, ci_low, ci_high) |>
  arrange(factor(level, levels = c("low", "mean", "high")), quantity)
write_csv(table_s6, file.path(output_dir, "table_S6_conditional_associations.csv"))

# Weighted descriptive statistics and correlations.
descriptive_vars <- c(
  T4STUD = "Teacher-student relations (X)",
  T4JOYTCH = "Joy of teaching (M)",
  T4JOBSAT = "Teacher job satisfaction (Y)",
  T4RELEAD = "Principal relational leadership (W)"
)

alpha_items <- list(
  T4STUD = paste0("TT4G65", LETTERS[1:4]),
  T4JOYTCH = paste0("TT4G80", LETTERS[1:4]),
  T4JOBSAT = paste0("TT4G78", c("A", "B", "C", "D", "E", "G", "J")),
  T4RELEAD = paste0("TT4G66", LETTERS[5:8])
)

alpha_source <- raw_codes[analytic_flag, , drop = FALSE]
for (nm in unlist(alpha_items, use.names = FALSE)) {
  alpha_source[[nm]][alpha_source[[nm]] %in% c(8, 9)] <- NA_real_
}
# Convert user-missing codes before reversing the two negatively worded items.
# Reversing first turns 8/9 into -3/-4, which are no longer recognized as
# missing and biases the seven-item descriptive alpha downward.
alpha_source$TT4G78C <- 5 - alpha_source$TT4G78C
alpha_source$TT4G78D <- 5 - alpha_source$TT4G78D

alphas <- vapply(
  names(alpha_items),
  function(scale) {
    weighted_alpha(
      alpha_source[alpha_items[[scale]]],
      analysis$TCHWGT
    )
  },
  numeric(1)
)

table2 <- data.frame(
  variable = names(descriptive_vars),
  label = unname(descriptive_vars),
  weighted_mean = vapply(
    names(descriptive_vars),
    function(v) wmean(analysis[[v]], analysis$TCHWGT),
    numeric(1)
  ),
  weighted_sd = vapply(
    names(descriptive_vars),
    function(v) wsd_pop(analysis[[v]], analysis$TCHWGT),
    numeric(1)
  ),
  weighted_alpha = unname(alphas[names(descriptive_vars)]),
  stringsAsFactors = FALSE
)
for (v in names(descriptive_vars)) {
  table2[[paste0("r_", v)]] <- vapply(
    names(descriptive_vars),
    function(v2) wcor(analysis[[v]], analysis[[v2]], analysis$TCHWGT),
    numeric(1)
  )
}
write_csv(table2, file.path(output_dir, "table_2_descriptives_correlations.csv"))

# Sample derivation and weighted demographics.
school_summary <- function(flag, stage, notes) {
  school_ids <- dat$IDSCHOOL[flag]
  counts <- table(school_ids)
  data.frame(
    stage = stage,
    unweighted_n = sum(flag),
    schools = length(counts),
    min_teachers_per_school = min(counts),
    max_teachers_per_school = max(counts),
    mean_teachers_per_school = mean(counts),
    notes = notes,
    stringsAsFactors = FALSE
  )
}

table1_flow <- bind_rows(
  school_summary(
    rep(TRUE, nrow(dat)),
    "Shanghai (China) ISCED 2 teacher file",
    "Forms A to C"
  ),
  school_summary(
    form_a,
    "Eligible Form A respondents",
    "Questionnaire Form A"
  ),
  school_summary(
    scale_complete,
    "All four official OECD scores available",
    "Five unavailable official-score intersections"
  ),
  school_summary(
    analytic_flag,
    "Analytic sample",
    "Sixteen incomplete controls after scale screening"
  )
)
write_csv(table1_flow, file.path(output_dir, "table_1_panel_A_sample_flow.csv"))

demographic_specs <- list(
  gender = list(
    source = "TT4G01",
    categories = list(Female = 1, Male = 2)
  ),
  age = list(
    source = "T4TAGEGR",
    categories = list(
      "Under 30 years" = 1,
      "30 to 39 years" = 2,
      "40 to 49 years" = 3,
      "50 years or older" = c(4, 5)
    )
  ),
  education = list(
    source = "T4THEDAT",
    categories = list(
      "ISCED 6 or below" = c(1, 2, 3),
      "ISCED 7 or 8" = c(4, 5)
    )
  ),
  experience = list(
    source = "T4TYEXPTT",
    categories = list(
      "5 years or less" = 1,
      "6 to 10 years" = 2,
      "11 to 20 years" = 3,
      "More than 20 years" = 4
    )
  )
)

weighted_category_rows <- function(data, w, group_name, source, categories) {
  bind_rows(lapply(names(categories), function(category) {
    flag <- data[[source]] %in% categories[[category]]
    data.frame(
      characteristic = group_name,
      category = category,
      unweighted_n = sum(flag, na.rm = TRUE),
      weighted_percent = 100 * sum(w[flag], na.rm = TRUE) / sum(w[!is.na(data[[source]])]),
      stringsAsFactors = FALSE
    )
  }))
}

table1_demo <- bind_rows(lapply(names(demographic_specs), function(group_name) {
  spec <- demographic_specs[[group_name]]
  weighted_category_rows(
    analysis,
    analysis$TCHWGT,
    group_name,
    spec$source,
    spec$categories
  )
}))
write_csv(table1_demo, file.path(output_dir, "table_1_panel_B_demographics.csv"))

# Table S1: distinguish structural non-administration from ordinary missingness.
s1_specs <- data.frame(
  variable = c(
    "T4STUD", "T4JOYTCH", "T4JOBSAT", "T4RELEAD",
    "TT4G01", "T4TAGEGR", "T4THEDAT", "T4TYEXPTT"
  ),
  description = c(
    "Teacher-student relations",
    "Joy of teaching",
    "Job satisfaction, overall",
    "Relational leadership",
    "Gender",
    "Age group",
    "Highest educational attainment",
    "Total teaching experience"
  ),
  administered_forms = c(
    "A and C", "A and B", "A, B, and C", "A and C",
    "A, B, and C", "A, B, and C", "A and B", "A, B, and C"
  ),
  planned_code = c(9998, 9998, 9998, 9998, 8, 8, 8, 8),
  ordinary_code = c(9999, 9999, 9999, 9999, 9, 9, 9, 9),
  stringsAsFactors = FALSE
)
s1_rows <- lapply(seq_len(nrow(s1_specs)), function(i) {
  spec <- s1_specs[i, ]
  vals <- raw_codes[[spec$variable]]
  data.frame(
    variable = spec$variable,
    description = spec$description,
    administered_forms = spec$administered_forms,
    planned_not_administered_full_n = sum(vals == spec$planned_code, na.rm = TRUE),
    ordinary_missing_full_n = sum(vals == spec$ordinary_code, na.rm = TRUE),
    ordinary_missing_form_A_n = sum(form_a & vals == spec$ordinary_code, na.rm = TRUE),
    valid_form_A_n = sum(form_a & !vals %in% c(spec$planned_code, spec$ordinary_code)),
    stringsAsFactors = FALSE
  )
})
table_s1 <- bind_rows(s1_rows)
write_csv(table_s1, file.path(output_dir, "table_S1_rotation_missingness.csv"))

# Table S2: included versus not-included Form A respondents.
form_a_included <- analytic_flag
form_a_not_included <- form_a & !analytic_flag

comparison_category_rows <- function(source, characteristic, categories) {
  bind_rows(lapply(names(categories), function(category) {
    values <- dat[[source]]
    category_values <- categories[[category]]
    included_valid <- form_a_included & !is.na(values)
    excluded_valid <- form_a_not_included & !is.na(values)
    in_cat <- values %in% category_values
    included_pct <- 100 *
      sum(dat$TCHWGT[included_valid & in_cat]) /
      sum(dat$TCHWGT[included_valid])
    excluded_pct <- 100 *
      sum(dat$TCHWGT[excluded_valid & in_cat]) /
      sum(dat$TCHWGT[excluded_valid])
    data.frame(
      characteristic = characteristic,
      category = category,
      included_n = sum(included_valid & in_cat),
      included_weighted_percent = included_pct,
      not_included_n = sum(excluded_valid & in_cat),
      not_included_weighted_percent = excluded_pct,
      difference_percentage_points = included_pct - excluded_pct,
      stringsAsFactors = FALSE
    )
  }))
}

table_s2 <- bind_rows(lapply(names(demographic_specs), function(characteristic) {
  spec <- demographic_specs[[characteristic]]
  comparison_category_rows(
    spec$source,
    characteristic,
    spec$categories
  )
}))
write_csv(table_s2, file.path(output_dir, "table_S2_included_comparison.csv"))

# Table S3: school sizes, ICC(1), and approximate design effects.
icc_oneway <- function(y, cluster) {
  ok <- is.finite(y) & !is.na(cluster)
  y <- y[ok]
  cluster <- factor(cluster[ok])
  n_j <- as.numeric(table(cluster))
  means_j <- tapply(y, cluster, mean)
  grand <- mean(y)
  ss_between <- sum(n_j * (means_j - grand)^2)
  ss_within <- sum((y - means_j[cluster])^2)
  j <- length(n_j)
  n <- length(y)
  ms_between <- ss_between / (j - 1)
  ms_within <- ss_within / (n - j)
  n0 <- (n - sum(n_j^2) / n) / (j - 1)
  (ms_between - ms_within) / (ms_between + (n0 - 1) * ms_within)
}

cluster_counts <- table(analysis$IDSCHOOL)
effective_mean_cluster <- nrow(analysis) / length(cluster_counts)
table_s3 <- data.frame(
  official_scale = scale_vars,
  teachers_n = nrow(analysis),
  schools_n = length(cluster_counts),
  ICC1 = vapply(
    scale_vars,
    function(v) icc_oneway(analysis[[v]], analysis$IDSCHOOL),
    numeric(1)
  ),
  effective_mean_cluster_size = effective_mean_cluster,
  stringsAsFactors = FALSE
)
table_s3$approximate_design_effect <- 1 +
  (table_s3$effective_mean_cluster_size - 1) * table_s3$ICC1
write_csv(table_s3, file.path(output_dir, "table_S3_clustering.csv"))

# Raw item means and the N=1,311 common sample.
common_items <- c(
  paste0("TT4G65", LETTERS[1:4]),
  paste0("TT4G80", LETTERS[1:4]),
  paste0("TT4G66", LETTERS[5:8]),
  paste0("TT4G78", c("A", "B", "E", "G", "H", "J"))
)
common_flag <- form_a & complete.cases(dat[c(common_items, control_vars)])
if (sum(common_flag) != 1311L) {
  stop("Common item-complete sample mismatch: ", sum(common_flag))
}

common <- dat[common_flag, , drop = FALSE]
common$male <- as.integer(common$TT4G01 == 2)
common$age30_39 <- as.integer(common$T4TAGEGR == 2)
common$age40_49 <- as.integer(common$T4TAGEGR == 3)
common$age50plus <- as.integer(common$T4TAGEGR %in% c(4, 5))
common$edu78 <- as.integer(common$T4THEDAT %in% c(4, 5))
common$exp6_10 <- as.integer(common$T4TYEXPTT == 2)
common$exp11_20 <- as.integer(common$T4TYEXPTT == 3)
common$exp20plus <- as.integer(common$T4TYEXPTT == 4)

common$X_raw <- rowMeans(common[paste0("TT4G65", LETTERS[1:4])])
common$M_raw <- rowMeans(common[paste0("TT4G80", LETTERS[1:4])])
common$W_raw <- rowMeans(common[paste0("TT4G66", LETTERS[5:8])])
common$Y_raw6 <- rowMeans(data.frame(
  A = common$TT4G78A,
  B = common$TT4G78B,
  E = common$TT4G78E,
  G = common$TT4G78G,
  H = common$TT4G78H,
  J = common$TT4G78J
))

add_centered <- function(d, x, m, w) {
  d$Xc <- d[[x]] - wmean(d[[x]], d$TCHWGT)
  d$Mc <- d[[m]] - wmean(d[[m]], d$TCHWGT)
  d$Wc <- d[[w]] - wmean(d[[w]], d$TCHWGT)
  d
}

common_official <- add_centered(common, "T4STUD", "T4JOYTCH", "T4RELEAD")
common_raw <- add_centered(common, "X_raw", "M_raw", "W_raw")

generic_model15_estimator <- function(outcome) {
  force(outcome)
  function(weights, d) {
    controls <- paste(control_terms, collapse = " + ")
    mediator_fit <- weighted_lm(
      as.formula(paste("Mc ~ Xc +", controls)), d, weights
    )
    outcome_fit <- weighted_lm(
      as.formula(
        paste(
          outcome, "~ Xc + Mc + Wc + Xc:Wc + Mc:Wc +",
          controls
        )
      ),
      d,
      weights
    )
    a <- coef_named(mediator_fit, "Xc")
    xw <- coef_named(outcome_fit, "Xc:Wc")
    mw <- coef_named(outcome_fit, "Mc:Wc")
    c(X_by_W = xw, M_by_W = mw, index_a_b3 = a * mw)
  }
}

write_log("Running sensitivity specifications for Table S5")
sens_max <- model15_brr$summary |>
  filter(metric %in% c("X_by_W", "M_by_W", "index_a_b3")) |>
  mutate(specification = "Official TALIS scores, maximum available sample (N=1338)")

sens_common <- brr_bundle(
  common_official,
  generic_model15_estimator("T4JOBSAT"),
  "Official common sample"
)$summary |>
  mutate(specification = "Official TALIS scores, common sample (N=1311)")

sens_raw <- brr_bundle(
  common_raw,
  generic_model15_estimator("Y_raw6"),
  "Raw item means"
)$summary |>
  mutate(specification = "Raw item means, common sample (N=1311)")

table_s5_panel_a <- bind_rows(sens_max, sens_common, sens_raw) |>
  select(specification, metric, estimate, se, t, df, p, ci_low, ci_high)
write_csv(
  table_s5_panel_a,
  file.path(output_dir, "table_S5_panel_A_specifications.csv")
)

panel_b_outcomes <- c(
  T4JOBSAT = "Overall job satisfaction",
  T4JSPROT = "Satisfaction with the profession",
  T4JSENVT = "Satisfaction with the work environment",
  Y_raw6 = "Six-item outcome mean used previously"
)
table_s5_panel_b <- bind_rows(lapply(names(panel_b_outcomes), function(outcome) {
  b <- brr_bundle(
    common_official,
    generic_model15_estimator(outcome),
    panel_b_outcomes[[outcome]]
  )$summary
  b |>
    filter(metric %in% c("M_by_W", "index_a_b3")) |>
    mutate(outcome = panel_b_outcomes[[outcome]])
}))
write_csv(
  table_s5_panel_b |>
    select(outcome, metric, estimate, se, t, df, p, ci_low, ci_high),
  file.path(output_dir, "table_S5_panel_B_outcomes.csv")
)

# Paired standardized contrast between profession and work-environment outcomes.
common_standardized <- common_official
common_standardized$Y_prof_z <- (
  common_standardized$T4JSPROT -
    wmean(common_standardized$T4JSPROT, common_standardized$TCHWGT)
) / wsd_pop(common_standardized$T4JSPROT, common_standardized$TCHWGT)
common_standardized$Y_env_z <- (
  common_standardized$T4JSENVT -
    wmean(common_standardized$T4JSENVT, common_standardized$TCHWGT)
) / wsd_pop(common_standardized$T4JSENVT, common_standardized$TCHWGT)

# The table reports the outcome-standardized contrast after putting the M-by-W
# product on weighted-SD units. Apply the same fixed scale to its associated
# index so that both rows use the manuscript's published metric.
contrast_scale <-
  wsd_pop(common_standardized$T4JOYTCH, common_standardized$TCHWGT) *
  wsd_pop(common_standardized$T4RELEAD, common_standardized$TCHWGT)

paired_contrast_estimator <- function(weights, d) {
  prof <- generic_model15_estimator("Y_prof_z")(weights, d)
  env <- generic_model15_estimator("Y_env_z")(weights, d)
  c(
    M_by_W_profession_minus_environment =
      contrast_scale * (prof[["M_by_W"]] - env[["M_by_W"]]),
    index_profession_minus_environment =
      contrast_scale * (prof[["index_a_b3"]] - env[["index_a_b3"]])
  )
}
paired_contrast <- brr_bundle(
  common_standardized,
  paired_contrast_estimator,
  "Paired standardized contrast"
)
write_csv(
  paired_contrast$summary,
  file.path(output_dir, "table_S5_paired_standardized_contrast.csv")
)

# Table S4: conventional OLS, weighted school-CR1, and Fay-BRR.
model4_fits_for_weights <- function(d, weights) {
  controls <- paste(control_terms, collapse = " + ")
  list(
    total = weighted_lm(as.formula(paste("T4JOBSAT ~ Xc +", controls)), d, weights),
    mediator = weighted_lm(as.formula(paste("T4JOYTCH ~ Xc +", controls)), d, weights),
    outcome = weighted_lm(as.formula(paste("T4JOBSAT ~ Xc + Mc +", controls)), d, weights)
  )
}

model15_fits_for_weights <- function(d, weights) {
  controls <- paste(control_terms, collapse = " + ")
  list(
    mediator = weighted_lm(as.formula(paste("T4JOYTCH ~ Xc +", controls)), d, weights),
    outcome = weighted_lm(
      as.formula(
        paste(
          "T4JOBSAT ~ Xc + Mc + Wc + Xc:Wc + Mc:Wc +",
          controls
        )
      ),
      d,
      weights
    )
  )
}

method_estimates <- function(d, weights) {
  m4 <- model4_fits_for_weights(d, weights)
  m15 <- model15_fits_for_weights(d, weights)
  a <- coef_named(m4$mediator, "Xc")
  b <- coef_named(m4$outcome, "Mc")
  b3 <- coef_named(m15$outcome, "Mc:Wc")
  c(
    a = a,
    b = b,
    total_c = coef_named(m4$total, "Xc"),
    direct_cprime = coef_named(m4$outcome, "Xc"),
    indirect_ab = a * b,
    X_by_W = coef_named(m15$outcome, "Xc:Wc"),
    M_by_W = b3,
    index_a_b3 = a * b3
  )
}

ols_weights <- rep(1, nrow(analysis))
ols_est <- method_estimates(analysis, ols_weights)
weighted_est <- method_estimates(analysis, analysis$TCHWGT)

ols_m4 <- model4_fits_for_weights(analysis, ols_weights)
ols_m15 <- model15_fits_for_weights(analysis, ols_weights)
ols_se <- c(
  a = sqrt(vcov(ols_m4$mediator)["Xc", "Xc"]),
  b = sqrt(vcov(ols_m4$outcome)["Mc", "Mc"]),
  total_c = sqrt(vcov(ols_m4$total)["Xc", "Xc"]),
  direct_cprime = sqrt(vcov(ols_m4$outcome)["Xc", "Xc"]),
  X_by_W = sqrt(vcov(ols_m15$outcome)["Xc:Wc", "Xc:Wc"]),
  M_by_W = sqrt(vcov(ols_m15$outcome)["Mc:Wc", "Mc:Wc"])
)

cross_equation_model_cov <- function(fit1, term1, fit2, term2) {
  x1 <- model.matrix(fit1)
  x2 <- model.matrix(fit2)
  e1 <- residuals(fit1)
  e2 <- residuals(fit2)
  w1 <- weights(fit1)
  w2 <- weights(fit2)
  if (is.null(w1)) w1 <- rep(1, length(e1))
  if (is.null(w2)) w2 <- rep(1, length(e2))
  common_rows <- intersect(as.integer(names(e1)), as.integer(names(e2)))
  i1 <- match(common_rows, as.integer(names(e1)))
  i2 <- match(common_rows, as.integer(names(e2)))
  sigma12 <- sum(
    sqrt(w1[i1] * w2[i2]) * e1[i1] * e2[i2]
  ) / sqrt(df.residual(fit1) * df.residual(fit2))
  b1 <- solve(crossprod(x1, x1 * w1))
  b2 <- solve(crossprod(x2, x2 * w2))
  cross_x <- crossprod(
    x1[i1, , drop = FALSE] * sqrt(w1[i1]),
    x2[i2, , drop = FALSE] * sqrt(w2[i2])
  )
  cross_v <- sigma12 * b1 %*% cross_x %*% b2
  cross_v[term1, term2]
}

ols_cov_a_b <- cross_equation_model_cov(
  ols_m4$mediator, "Xc", ols_m4$outcome, "Mc"
)
ols_cov_a_b3 <- cross_equation_model_cov(
  ols_m15$mediator, "Xc", ols_m15$outcome, "Mc:Wc"
)
ols_se[["indirect_ab"]] <- sqrt(
  ols_est[["b"]]^2 * ols_se[["a"]]^2 +
    ols_est[["a"]]^2 * ols_se[["b"]]^2 +
    2 * ols_est[["a"]] * ols_est[["b"]] * ols_cov_a_b
)
ols_se[["index_a_b3"]] <- sqrt(
  ols_est[["M_by_W"]]^2 * ols_se[["a"]]^2 +
    ols_est[["a"]]^2 * ols_se[["M_by_W"]]^2 +
    2 * ols_est[["a"]] * ols_est[["M_by_W"]] * ols_cov_a_b3
)

weighted_m4 <- model4_fits_for_weights(analysis, analysis$TCHWGT)
weighted_m15 <- model15_fits_for_weights(analysis, analysis$TCHWGT)
cr1_single_se <- c(
  a = sqrt(vcovCL(
    weighted_m4$mediator,
    cluster = analysis$IDSCHOOL,
    type = "HC1"
  )["Xc", "Xc"]),
  b = sqrt(vcovCL(
    weighted_m4$outcome,
    cluster = analysis$IDSCHOOL,
    type = "HC1"
  )["Mc", "Mc"]),
  total_c = sqrt(vcovCL(
    weighted_m4$total,
    cluster = analysis$IDSCHOOL,
    type = "HC1"
  )["Xc", "Xc"]),
  direct_cprime = sqrt(vcovCL(
    weighted_m4$outcome,
    cluster = analysis$IDSCHOOL,
    type = "HC1"
  )["Xc", "Xc"]),
  X_by_W = sqrt(vcovCL(
    weighted_m15$outcome,
    cluster = analysis$IDSCHOOL,
    type = "HC1"
  )["Xc:Wc", "Xc:Wc"]),
  M_by_W = sqrt(vcovCL(
    weighted_m15$outcome,
    cluster = analysis$IDSCHOOL,
    type = "HC1"
  )["Mc:Wc", "Mc:Wc"])
)

cluster_cross_cov <- function(fit1, term1, fit2, term2, cluster) {
  x1 <- model.matrix(fit1)
  x2 <- model.matrix(fit2)
  e1 <- residuals(fit1)
  e2 <- residuals(fit2)
  w1 <- weights(fit1)
  w2 <- weights(fit2)
  rows1 <- as.integer(names(e1))
  rows2 <- as.integer(names(e2))
  common_rows <- intersect(rows1, rows2)
  i1 <- match(common_rows, rows1)
  i2 <- match(common_rows, rows2)
  # `cluster` is in the same order as fit1's model frame. Residual names retain
  # source-file row names after subsetting, so they must not be used as numeric
  # positions into the shorter analysis vector.
  cl <- cluster[i1]
  bread1 <- solve(crossprod(x1, x1 * w1))
  bread2 <- solve(crossprod(x2, x2 * w2))
  clusters <- unique(cl)
  infl1 <- matrix(0, nrow = length(clusters), ncol = ncol(x1))
  infl2 <- matrix(0, nrow = length(clusters), ncol = ncol(x2))
  colnames(infl1) <- colnames(x1)
  colnames(infl2) <- colnames(x2)
  for (g in seq_along(clusters)) {
    idx <- which(cl == clusters[[g]])
    score1 <- colSums(x1[i1[idx], , drop = FALSE] * (w1[i1[idx]] * e1[i1[idx]]))
    score2 <- colSums(x2[i2[idx], , drop = FALSE] * (w2[i2[idx]] * e2[i2[idx]]))
    infl1[g, ] <- bread1 %*% score1
    infl2[g, ] <- bread2 %*% score2
  }
  n <- length(common_rows)
  g <- length(clusters)
  adj1 <- (g / (g - 1)) * ((n - 1) / (n - ncol(x1)))
  adj2 <- (g / (g - 1)) * ((n - 1) / (n - ncol(x2)))
  cross <- sqrt(adj1 * adj2) * crossprod(infl1, infl2)
  cross[term1, term2]
}

cr1_cov_a_b <- cluster_cross_cov(
  weighted_m4$mediator, "Xc",
  weighted_m4$outcome, "Mc",
  analysis$IDSCHOOL
)
cr1_cov_a_b3 <- cluster_cross_cov(
  weighted_m15$mediator, "Xc",
  weighted_m15$outcome, "Mc:Wc",
  analysis$IDSCHOOL
)
cr1_single_se[["indirect_ab"]] <- sqrt(
  weighted_est[["b"]]^2 * cr1_single_se[["a"]]^2 +
    weighted_est[["a"]]^2 * cr1_single_se[["b"]]^2 +
    2 * weighted_est[["a"]] * weighted_est[["b"]] * cr1_cov_a_b
)
cr1_single_se[["index_a_b3"]] <- sqrt(
  weighted_est[["M_by_W"]]^2 * cr1_single_se[["a"]]^2 +
    weighted_est[["a"]]^2 * cr1_single_se[["M_by_W"]]^2 +
    2 * weighted_est[["a"]] * weighted_est[["M_by_W"]] * cr1_cov_a_b3
)

fay_lookup <- bind_rows(
  model4_brr$summary |>
    filter(metric %in% c("a", "b", "total_c", "direct_cprime", "indirect_ab")),
  model15_brr$summary |>
    filter(metric %in% c("X_by_W", "M_by_W", "index_a_b3"))
) |>
  select(metric, estimate, se, ci_low, ci_high, p)

make_method_rows <- function(method, estimates, ses, df) {
  metrics <- names(estimates)
  data.frame(
    method = method,
    metric = metrics,
    estimate = unname(estimates),
    se = unname(ses[metrics]),
    df = df,
    t = unname(estimates / ses[metrics]),
    p = 2 * pt(-abs(estimates / ses[metrics]), df = df),
    ci_low = unname(estimates - qt(0.975, df) * ses[metrics]),
    ci_high = unname(estimates + qt(0.975, df) * ses[metrics]),
    stringsAsFactors = FALSE
  )
}

table_s4 <- bind_rows(
  make_method_rows("Unweighted OLS", ols_est, ols_se, df.residual(ols_m15$outcome)),
  make_method_rows("Weighted school CR1", weighted_est, cr1_single_se, 204),
  fay_lookup |>
    mutate(method = "Weighted Fay-BRR", df = 99, t = estimate / se) |>
    select(method, metric, estimate, se, df, t, p, ci_low, ci_high)
)
write_csv(table_s4, file.path(output_dir, "table_S4_inference_methods.csv"))

# Hypothesis summary (Table S7).
lookup <- function(bundle, metric) {
  bundle$summary[bundle$summary$metric == metric, , drop = FALSE]
}
table_s7 <- data.frame(
  hypothesis = paste0("H", 1:6),
  statistical_test = c(
    sprintf("c = %.4f, p = %.4g", lookup(model4_brr, "total_c")$estimate, lookup(model4_brr, "total_c")$p),
    sprintf("a = %.4f, p = %.4g", lookup(model4_brr, "a")$estimate, lookup(model4_brr, "a")$p),
    sprintf("b = %.4f, p = %.4g", lookup(model4_brr, "b")$estimate, lookup(model4_brr, "b")$p),
    sprintf(
      "a*b = %.4f, 95%% CI [%.4f, %.4f]",
      lookup(model4_brr, "indirect_ab")$estimate,
      lookup(model4_brr, "indirect_ab")$ci_low,
      lookup(model4_brr, "indirect_ab")$ci_high
    ),
    sprintf(
      "X*W = %.4f, p = %.4g",
      lookup(model15_brr, "X_by_W")$estimate,
      lookup(model15_brr, "X_by_W")$p
    ),
    sprintf(
      "M*W = %.4f, p = %.4g; index = %.4f, p = %.4g",
      lookup(model15_brr, "M_by_W")$estimate,
      lookup(model15_brr, "M_by_W")$p,
      lookup(model15_brr, "index_a_b3")$estimate,
      lookup(model15_brr, "index_a_b3")$p
    )
  ),
  decision = c(
    "Supported",
    "Supported",
    "Supported",
    "Supported as a statistical indirect association",
    "Not supported",
    paste(
      "Supported in the declared official-score primary model,",
      "but measurement-sensitive and exploratory"
    )
  ),
  stringsAsFactors = FALSE
)
write_csv(table_s7, file.path(output_dir, "table_S7_hypotheses.csv"))

# Weighted R-squared values and interaction Wald test.
primary_m4_fits <- model4_fits_for_weights(analysis, analysis$TCHWGT)
primary_m15_fits <- model15_fits_for_weights(analysis, analysis$TCHWGT)
r_squared <- data.frame(
  model = c(
    "Model 4 total association",
    "Model 4 mediator equation",
    "Model 4 outcome equation",
    "Model 15 outcome equation"
  ),
  weighted_R2 = c(
    summary(primary_m4_fits$total)$r.squared,
    summary(primary_m4_fits$mediator)$r.squared,
    summary(primary_m4_fits$outcome)$r.squared,
    summary(primary_m15_fits$outcome)$r.squared
  )
)
write_csv(r_squared, file.path(output_dir, "model_fit_descriptive.csv"))

interaction_terms <- c("X_by_W", "M_by_W")
idx <- match(interaction_terms, rownames(model15_brr$covariance))
interaction_beta <- model15_brr$point[interaction_terms]
interaction_cov <- model15_brr$covariance[idx, idx, drop = FALSE]
wald_chisq <- as.numeric(t(interaction_beta) %*% solve(interaction_cov) %*% interaction_beta)
wald <- data.frame(
  test = "Joint Wald test of X*W and M*W",
  statistic = wald_chisq,
  numerator_df = 2,
  denominator_df = 99,
  p_F = pf(wald_chisq / 2, 2, 99, lower.tail = FALSE),
  p_chisq = pchisq(wald_chisq, 2, lower.tail = FALSE)
)
write_csv(wald, file.path(output_dir, "interaction_wald_test.csv"))

# Figure 3: predictions at low/high joy of teaching and three W levels.
outcome15 <- primary_m15_fits$outcome
joy_values <- c(
  low = center_constants[["M"]] -
    wsd_pop(analysis$T4JOYTCH, analysis$TCHWGT),
  high = center_constants[["M"]] +
    wsd_pop(analysis$T4JOYTCH, analysis$TCHWGT)
)
prediction_grid <- expand.grid(
  joy_level = names(joy_values),
  leadership_level = names(probe_original),
  stringsAsFactors = FALSE
)
prediction_grid$T4JOYTCH <- unname(joy_values[prediction_grid$joy_level])
prediction_grid$T4RELEAD <- unname(probe_original[prediction_grid$leadership_level])
prediction_grid$Xc <- 0
prediction_grid$Mc <- prediction_grid$T4JOYTCH - center_constants[["M"]]
prediction_grid$Wc <- prediction_grid$T4RELEAD - center_constants[["W"]]
for (term in control_terms) prediction_grid[[term]] <- 0
prediction_grid$predicted_job_satisfaction <- predict(outcome15, newdata = prediction_grid)
write_csv(prediction_grid, file.path(output_dir, "figure_3_plot_data.csv"))

figure3 <- ggplot(
  prediction_grid,
  aes(
    x = T4JOYTCH,
    y = predicted_job_satisfaction,
    color = leadership_level,
    group = leadership_level
  )
) +
  geom_line(linewidth = 0.9) +
  geom_point(size = 2.5) +
  scale_color_manual(
    values = c(low = "#0072B2", mean = "#555555", high = "#D55E00"),
    breaks = c("low", "mean", "high"),
    labels = c("Low leadership", "Mean leadership", "High leadership")
  ) +
  labs(
    x = "Joy of teaching (official TALIS scale)",
    y = "Predicted overall job satisfaction",
    color = "Principal relational leadership"
  ) +
  theme_classic(base_size = 11) +
  theme(legend.position = "top")
ggsave(
  filename = file.path(output_dir, "figure_3_reproduced.png"),
  plot = figure3,
  width = 7,
  height = 4.8,
  dpi = 300
)

# Variable mapping derived from the original labels.
roles <- c(
  IDTQUEST = "Questionnaire form",
  IDSCHOOL = "School cluster ID",
  IDTEACH = "Teacher ID",
  TCHWGT = "Final teacher weight",
  setNames(rep("Fay-BRR replicate weight", 100), repweight_vars),
  T4STUD = "Independent variable (X)",
  T4JOYTCH = "Mediator (M)",
  T4JOBSAT = "Primary outcome (Y)",
  T4JSPROT = "Sensitivity outcome",
  T4JSENVT = "Sensitivity outcome",
  T4RELEAD = "Moderator (W)",
  TT4G01 = "Control: gender",
  T4TAGEGR = "Control: age group",
  T4THEDAT = "Control: education",
  T4TYEXPTT = "Control: teaching experience"
)
item_roles <- c(
  setNames(rep("T4STUD source item", 4), paste0("TT4G65", LETTERS[1:4])),
  setNames(rep("T4JOYTCH source item", 4), paste0("TT4G80", LETTERS[1:4])),
  setNames(rep("T4RELEAD source item", 4), paste0("TT4G66", LETTERS[5:8])),
  setNames(
    rep("Job-satisfaction source item", 8),
    paste0("TT4G78", c("A", "B", "C", "D", "E", "G", "H", "J"))
  )
)
roles <- c(roles, item_roles)
mapping_vars <- unique(names(roles))
variable_mapping <- data.frame(
  variable = mapping_vars,
  role = unname(roles[mapping_vars]),
  source_label = vapply(
    mapping_vars,
    function(v) {
      label <- attr(raw[[v]], "label")
      if (is.null(label)) "" else label
    },
    character(1)
  ),
  missing_codes = vapply(
    mapping_vars,
    function(v) {
      missing <- attr(raw[[v]], "na_values")
      if (is.null(missing)) "" else paste(missing, collapse = ";")
    },
    character(1)
  ),
  stringsAsFactors = FALSE
)
write_csv(variable_mapping, file.path(output_dir, "variable_mapping.csv"))

# Machine-readable validation against values printed in the submitted manuscript.
expected <- data.frame(
  source = c(
    rep("Table 3", 5),
    rep("Table 4", 6),
    rep("Table S3", 4),
    rep("Table S5 raw means", 3)
  ),
  metric = c(
    "total_c", "a", "b", "direct_cprime", "indirect_ab",
    "direct_at_mean_W", "M_to_Y_at_mean_W", "W_main",
    "X_by_W", "M_by_W", "index_a_b3",
    paste0("ICC_", scale_vars),
    "X_by_W", "M_by_W", "index_a_b3"
  ),
  expected_estimate = c(
    0.4371, 0.4631, 0.4603, 0.2239, 0.2132,
    0.0356, 0.4021, 0.3560, -0.0057, 0.0477, 0.0221,
    0.0571, 0.0738, 0.0890, 0.0919,
    0.0120, 0.1009, 0.0484
  ),
  stringsAsFactors = FALSE
)
observed <- c(
  setNames(model4_brr$summary$estimate, model4_brr$summary$metric),
  setNames(model15_brr$summary$estimate, model15_brr$summary$metric),
  setNames(table_s3$ICC1, paste0("ICC_", table_s3$official_scale)),
  setNames(
    sens_raw$estimate,
    paste0("raw_", sens_raw$metric)
  )
)
expected$observed_estimate <- vapply(seq_len(nrow(expected)), function(i) {
  key <- expected$metric[[i]]
  if (expected$source[[i]] == "Table S5 raw means") {
    key <- paste0("raw_", key)
  }
  unname(observed[[key]])
}, numeric(1))
expected$absolute_difference <- abs(
  expected$observed_estimate - expected$expected_estimate
)
expected$matches_reported_rounding <- expected$absolute_difference <= 0.00015
write_csv(expected, file.path(output_dir, "validation_against_manuscript.csv"))

# Input integrity and session information. Use operating-system tools so the
# published package does not add a non-statistical R-package dependency.
if (.Platform$OS.type == "windows") {
  hash_output <- system2(
    "certutil",
    c("-hashfile", shQuote(args[[1]]), "SHA256"),
    stdout = TRUE,
    stderr = TRUE
  )
} else {
  hash_output <- system2(
    "sha256sum",
    shQuote(input_path),
    stdout = TRUE,
    stderr = TRUE
  )
}
hash_output <- iconv(hash_output, from = "", to = "UTF-8", sub = "")
hash_matches <- regmatches(
  hash_output,
  regexpr("[[:xdigit:]]{64}", hash_output, perl = TRUE)
)
hash_matches <- hash_matches[nchar(hash_matches) == 64L]
if (!length(hash_matches)) {
  stop("Could not parse SHA-256 output.")
}
input_sha256 <- tolower(hash_matches[[1]])
writeLines(
  c(
    paste0("File: ", basename(input_path)),
    paste0("SHA256: ", input_sha256)
  ),
  file.path(output_dir, "input_sha256.txt"),
  useBytes = TRUE
)
capture.output(sessionInfo(), file = file.path(output_dir, "sessionInfo.txt"))

# Reproducibility status.
validation_status <- data.frame(
  check = c(
    "Rows",
    "Form A respondents",
    "Analytic sample",
    "Item-complete sample",
    "Schools in analytic sample",
    "Reported estimates matched at printed precision"
  ),
  observed = c(
    nrow(dat),
    sum(form_a),
    nrow(analysis),
    nrow(common),
    length(unique(analysis$IDSCHOOL)),
    sum(expected$matches_reported_rounding)
  ),
  expected = c(
    4078,
    1359,
    1338,
    1311,
    205,
    nrow(expected)
  ),
  passed = c(
    nrow(dat) == 4078,
    sum(form_a) == 1359,
    nrow(analysis) == 1338,
    nrow(common) == 1311,
    length(unique(analysis$IDSCHOOL)) == 205,
    all(expected$matches_reported_rounding)
  )
)
write_csv(validation_status, file.path(output_dir, "validation_status.csv"))

write_log(
  "Completed. Manuscript-precision matches: ",
  sum(expected$matches_reported_rounding),
  "/",
  nrow(expected)
)
write_log("Analysis finished successfully")
```

# **Appendix B. Variable mapping**

Every source variable read by the script, its role in the analysis, its source label in the TALIS teacher file, and the codes treated as missing.

| Variable | Role | Source label | Missing codes |
| --- | --- | --- | --- |
| IDTQUEST | Questionnaire form | Teacher Questionnaire Form Type | 8;9 |
| IDSCHOOL | School cluster ID | School ID | 9998;9999 |
| IDTEACH | Teacher ID | Teacher ID | 999998;999999 |
| TCHWGT | Final teacher weight | Teacher Final Weight | 9998;9999 |
| TRWGT1 to TRWGT100 | Fay-BRR replicate weights | Teacher BRR-Fay Replicate Weights 1 to 100 | 999998;999999 |
| T4STUD | Independent variable (X) | Teacher-student relations | 9998;9999 |
| T4JOYTCH | Mediator (M) | Joy of teaching | 9998;9999 |
| T4JOBSAT | Primary outcome (Y) | Job satisfaction, overall | 9998;9999 |
| T4JSPROT | Sensitivity outcome | Job satisfaction with profession | 9998;9999 |
| T4JSENVT | Sensitivity outcome | Job satisfaction with work environment | 9998;9999 |
| T4RELEAD | Moderator (W) | Relational leadership | 9998;9999 |
| TT4G01 | Control: gender | Background/ Gender | 8;9 |
| T4TAGEGR | Control: age group | Teacher age (grouped) | 8;9 |
| T4THEDAT | Control: education | Teacher's highest level of formal education completed (grouped) | 8;9 |
| T4TYEXPTT | Control: teaching experience | Teacher's years of work experience in total (grouped) | 8;9 |
| TT4G65A | T4STUD source item | School Climate/ Teacher-student relations/ Teachers and students usually get on well with each other | 8;9 |
| TT4G65B | T4STUD source item | School Climate/ Teacher-student relations/ Most teachers believe that the students’ well-being is important | 8;9 |
| TT4G65C | T4STUD source item | School Climate/ Teacher-student relations/ Most teachers are interested in what students have to say | 8;9 |
| TT4G65D | T4STUD source item | School Climate/ Teacher-student relations/ If a student needs extra assistance, the school provides it | 8;9 |
| TT4G80A | T4JOYTCH source item | Occupational Perceptions/ Enjoyment of teaching/ I like the subject(s) that I teach | 8;9 |
| TT4G80B | T4JOYTCH source item | Occupational Perceptions/ Enjoyment of teaching/ I often feel happy while I teach | 8;9 |
| TT4G80C | T4JOYTCH source item | Occupational Perceptions/ Enjoyment of teaching/ I generally teach with enthusiasm | 8;9 |
| TT4G80D | T4JOYTCH source item | Occupational Perceptions/ Enjoyment of teaching/ The interesting challenges of teaching give me satisfaction | 8;9 |
| TT4G66E | T4RELEAD source item | School Climate/ Perceptions about the principal/ Principal encourages all staff to have a say on important decisions | 8;9 |
| TT4G66F | T4RELEAD source item | School Climate/ Perceptions about the principal/ Principal has good professional relationships with staff | 8;9 |
| TT4G66G | T4RELEAD source item | School Climate/ Perceptions about the principal/ Principal has good professional relationships with parents or guardians | 8;9 |
| TT4G66H | T4RELEAD source item | School Climate/ Perceptions about the principal/ Principal has good professional relationships with students | 8;9 |
| TT4G78A | Job-satisfaction source item | Occupational Perceptions/ Job satisfaction/ The advantages clearly outweigh the disadvantages | 8;9 |
| TT4G78B | Job-satisfaction source item | Occupational Perceptions/ Job satisfaction/ If I could decide again, I would still choose to work as a teacher | 8;9 |
| TT4G78C | Job-satisfaction source item | Occupational Perceptions/ Job satisfaction/ I would like to change to another school if that were possible | 8;9 |
| TT4G78D | Job-satisfaction source item | Occupational Perceptions/ Job satisfaction/ I regret that I decided to become a teacher | 8;9 |
| TT4G78E | Job-satisfaction source item | Occupational Perceptions/ Job satisfaction/ I enjoy working at this school | 8;9 |
| TT4G78G | Job-satisfaction source item | Occupational Perceptions/ Job satisfaction/ I would recommend my school as a good place to work | 8;9 |
| TT4G78H | Job-satisfaction source item | Occupational Perceptions/ Job satisfaction/ I think that the teaching profession is valued in society | 8;9 |
| TT4G78J | Job-satisfaction source item | Occupational Perceptions/ Job satisfaction/ All in all, I am satisfied with my job | 8;9 |

# **Appendix C. Aggregate outputs**

## **C.1 Sample flow**

| Stage | n | Schools | Min | Max | Mean | Notes |
| --- | --- | --- | --- | --- | --- | --- |
| Shanghai (China) ISCED 2 teacher file | 4078 | 205 | 16 | 29 | 19.89 | Forms A to C |
| Eligible Form A respondents | 1359 | 205 | 4 | 10 | 6.63 | Questionnaire Form A |
| All four official OECD scores available | 1354 | 205 | 4 | 10 | 6.60 | Five unavailable official-score intersections |
| Analytic sample | 1338 | 205 | 4 | 9 | 6.53 | Sixteen incomplete controls after scale screening |

## **C.2 Demographics**

| Characteristic | Category | n | Weighted % |
| --- | --- | --- | --- |
| gender | Female | 984 | 74.19 |
| gender | Male | 354 | 25.81 |
| age | Under 30 years | 249 | 19.61 |
| age | 30 to 39 years | 413 | 30.82 |
| age | 40 to 49 years | 400 | 29.52 |
| age | 50 years or older | 276 | 20.05 |
| education | ISCED 6 or below | 1038 | 77.18 |
| education | ISCED 7 or 8 | 300 | 22.82 |
| experience | 5 years or less | 269 | 21.17 |
| experience | 6 to 10 years | 243 | 17.81 |
| experience | 11 to 20 years | 317 | 23.46 |
| experience | More than 20 years | 509 | 37.57 |

## **C.3 Descriptive statistics and correlations**

| Variable | M | SD | alpha | X | M | Y | W |
| --- | --- | --- | --- | --- | --- | --- | --- |
| Teacher-student relations (X) | 14.376 | 2.014 | 0.907 | 1.000 | 0.452 | 0.453 | 0.648 |
| Joy of teaching (M) | 13.412 | 2.082 | 0.909 | 0.452 | 1.000 | 0.599 | 0.418 |
| Teacher job satisfaction (Y) | 9.567 | 1.958 | 0.845 | 0.453 | 0.599 | 1.000 | 0.547 |
| Principal relational leadership (W) | 13.238 | 1.897 | 0.961 | 0.648 | 0.418 | 0.547 | 1.000 |

## **C.4 Model 4**

| Quantity | B | SE | t(99) | p | 95% CI |
| --- | --- | --- | --- | --- | --- |
| Total association c | 0.4371 | 0.0278 | 15.705 | < 0.001 | [0.3819, 0.4923] |
| X to M path a | 0.4631 | 0.0284 | 16.293 | < 0.001 | [0.4067, 0.5195] |
| M to Y path b | 0.4603 | 0.0286 | 16.094 | < 0.001 | [0.4036, 0.5171] |
| Direct association c' | 0.2239 | 0.0285 | 7.847 | < 0.001 | [0.1673, 0.2806] |
| Indirect association a x b | 0.2132 | 0.0193 | 11.027 | < 0.001 | [0.1748, 0.2515] |

## **C.5 Model 15**

| Quantity | B | SE | t(99) | p | 95% CI |
| --- | --- | --- | --- | --- | --- |
| Direct association at mean W | 0.0356 | 0.0297 | 1.201 | 0.2326 | [-0.0232, 0.0945] |
| M to Y at mean W | 0.4021 | 0.0288 | 13.937 | < 0.001 | [0.3448, 0.4593] |
| W main association | 0.3560 | 0.0317 | 11.235 | < 0.001 | [0.2931, 0.4188] |
| X x W | -0.0057 | 0.0112 | -0.512 | 0.6101 | [-0.0280, 0.0165] |
| M x W | 0.0477 | 0.0143 | 3.341 | 0.0012 | [0.0194, 0.0760] |
| Index of moderated mediation a x b3 | 0.0221 | 0.0069 | 3.222 | 0.0017 | [0.0085, 0.0357] |

## **C.6 Model fit and joint interaction test**

| Model | Weighted R-squared |
| --- | --- |
| Model 4 total association | 0.2185 |
| Model 4 mediator equation | 0.2185 |
| Model 4 outcome equation | 0.4058 |
| Model 15 outcome equation | 0.4772 |

Joint Wald test of X x W and M x W: F(2, 99) = 6.1840, p = 0.0029; Wald chi-square(2) = 12.3680, p = 0.0021.

## **C.7 Figure 3 plot data**

| Joy of teaching | Relational leadership | M (raw) | W (raw) | Predicted job satisfaction |
| --- | --- | --- | --- | --- |
| low | low | 11.330 | 11.341 | 8.1786 |
| high | low | 15.494 | 11.341 | 9.4761 |
| low | mean | 11.330 | 13.238 | 8.6657 |
| high | mean | 15.494 | 13.238 | 10.3399 |
| low | high | 11.330 | 15.135 | 9.1527 |
| high | high | 15.494 | 15.135 | 11.2037 |

*Reproduced Figure 3, as produced directly by the analysis script. The plotted values are identical to those in Figure 3 of the article; the article version has been redrawn to the journal's figure specifications.*

## **C.8 Validation against the manuscript**

| Source | Quantity | Reported | Reproduced | Abs. difference | Match |
| --- | --- | --- | --- | --- | --- |
| Table 3 | Total association c | 0.4371 | 0.437113 | 1.3e-5 | Yes |
| Table 3 | X to M path a | 0.4631 | 0.463108 | 7.7e-6 | Yes |
| Table 3 | M to Y path b | 0.4603 | 0.460307 | 7.3e-6 | Yes |
| Table 3 | Direct association c' | 0.2239 | 0.223941 | 4.1e-5 | Yes |
| Table 3 | Indirect association a x b | 0.2132 | 0.213172 | 2.8e-5 | Yes |
| Table 4 | Direct association at mean W | 0.0356 | 0.035625 | 2.5e-5 | Yes |
| Table 4 | M to Y at mean W | 0.4021 | 0.402051 | 4.9e-5 | Yes |
| Table 4 | W main association | 0.356 | 0.355973 | 2.7e-5 | Yes |
| Table 4 | X x W | -0.0057 | -0.005730 | 3.0e-5 | Yes |
| Table 4 | M x W | 0.0477 | 0.047682 | 1.8e-5 | Yes |
| Table 4 | Index of moderated mediation a x b3 | 0.0221 | 0.022082 | 1.8e-5 | Yes |
| Table S3 | ICC_T4STUD | 0.0571 | 0.057088 | 1.2e-5 | Yes |
| Table S3 | ICC_T4JOYTCH | 0.0738 | 0.073836 | 3.6e-5 | Yes |
| Table S3 | ICC_T4JOBSAT | 0.089 | 0.089001 | 7.8e-7 | Yes |
| Table S3 | ICC_T4RELEAD | 0.0919 | 0.091875 | 2.5e-5 | Yes |
| Table S5 raw means | X x W | 0.012 | 0.012038 | 3.8e-5 | Yes |
| Table S5 raw means | M x W | 0.1009 | 0.100859 | 4.1e-5 | Yes |
| Table S5 raw means | Index of moderated mediation a x b3 | 0.0484 | 0.048439 | 3.9e-5 | Yes |

**C.9 Verified implementation details**

The independent verification run resolved two implementation details in the archived script. SPSS user-missing codes were removed before reverse scoring the two negatively worded items, and the paired Table S5 contrast was expressed in the weighted-SD metric used in the S1 File.

| Check | Verified value | Manuscript/S1 value | Status |
| --- | --- | --- | --- |
| Seven-item weighted alpha | 0.845018 | 0.845 | MATCH |
| M x W: profession minus work environment | -0.059214; 95% CI [-0.111660, -0.006768]; p = 0.027309 | -0.0592; 95% CI [-0.1117, -0.0068]; p = 0.0273 | MATCH |
| Index: profession minus work environment | -0.027828; 95% CI [-0.052340, -0.003317]; p = 0.026486 | -0.0278; 95% CI [-0.0523, -0.0033]; p = 0.0265 | MATCH |

These corrections affect only the displayed descriptive alpha and the scale of the paired Table S5 contrast; they do not change the 18 core benchmark estimates, their test statistics, or the article's hypothesis decisions.

# **Appendix D. Software session information and input identity**

The output below is the sessionInfo() record captured during the independently verified corrected run on 18 August 2026.

```
R version 4.6.0 (2026-04-24 ucrt)
Platform: x86_64-w64-mingw32/x64
Running under: Windows 11 x64 (build 26200)

Matrix products: default
  LAPACK version 3.12.1

locale:
[1] C
system code page: 65001

time zone: America/Tijuana
tzcode source: internal

attached base packages:
[1] grid      stats     graphics  grDevices utils     datasets  methods
[8] base

other attached packages:
 [1] readr_2.2.0    tidyr_1.3.2    dplyr_1.2.1    ggplot2_4.0.3  psych_2.6.5
 [6] sandwich_3.1-1 survey_4.5     survival_3.8-6 Matrix_1.7-5   haven_2.5.5

loaded via a namespace (and not attached):
 [1] bit_4.6.0          gtable_0.3.6       crayon_1.5.3       compiler_4.6.0
 [5] tidyselect_1.2.1   Rcpp_1.1.1-1.1     parallel_4.6.0     textshaping_1.0.5
 [9] systemfonts_1.3.2  splines_4.6.0      scales_1.4.0       lattice_0.22-9
[13] R6_2.6.1           labeling_0.4.3     generics_0.1.4     forcats_1.0.1
[17] tibble_3.3.1       DBI_1.3.0          tzdb_0.5.0         pillar_1.11.1
[21] RColorBrewer_1.1-3 rlang_1.2.0        S7_0.2.2           bit64_4.8.2
[25] otel_0.2.0         cli_3.6.6          withr_3.0.2        magrittr_2.0.5
[29] vroom_1.7.1        hms_1.1.4          lifecycle_1.0.5    nlme_3.1-169
[33] vctrs_0.7.3        mnormt_2.1.2       glue_1.8.1         farver_2.1.2
[37] ragg_1.5.2         zoo_1.8-15         mitools_2.4        purrr_1.2.2
[41] tools_4.6.0        pkgconfig_2.0.3
```

## **Input file identity**

File: TALIS2024_BTG_CSH_teacher.sav

SHA256: b4f63148a6eba46ef8f88619b834f660414767cc3f860857faa156953356b7fc
